# Transplantation of Skeletal Muscle-Derived Sca-1^+^/PW1^+^/Pax7^−^ Interstitial Cells (PICs) Improves Cardiac Function and Attenuates Remodeling in Mice Subjected to Myocardial Infarction

**DOI:** 10.3390/cells11244050

**Published:** 2022-12-12

**Authors:** Prashant J. Ruchaya, Fiona C. Lewis-McDougall, Nitiphat Sornkarn, Sachin Amin, Benjamin Grimsdell, Abeer Shaalan, Guilia Gritti, Kyi Thar Soe, James E. Clark, Georgina M. Ellison-Hughes

**Affiliations:** 1 Centre for Human and Applied Physiological Sciences, School of Basic and Medical Biosciences, Faculty of Life Sciences & Medicine, King’s College London, Guy’s Campus, London SE1 1UL, UK; f.lewis@qmul.ac.uk (F.C.L.-M.); nitiphat.sornkarn@kcl.ac.uk (N.S.); sachin.amin@kcl.ac.uk (S.A.); benjamin.grimsdell@kcl.ac.uk (B.G.); shaalan.abeer@kcl.ac.uk (A.S.); g.gritti@kcl.ac.uk (G.G.); 2 Centre for Gene Therapy and Regenerative Medicine, School of Basic and Medical Biosciences, Faculty of Life Sciences & Medicine, King’s College London, Guy’s Campus, London SE1 1UL, UK; 3 School of Health, Sport and Biosciences, Stratford Campus, University of East London, London E16 2RD, UK; u1819492@uel.ac.uk; 4 The William Harvey Research Institute, Charterhouse Square, Barts & The London School of Medicine & Dentistry, Queen Mary University of London, London EC1M 6BQ, UK; 5 Rayne Institute, School of Cardiovascular and Metabolic Medicine and Sciences, Faculty of Life Sciences & Medicine, King’s College London, St Thomas’ Campus, London SE1 7EH, UK; james.2.clark@kcl.ac.uk

**Keywords:** skeletal muscle PW1^+^/Pax7^−^ interstitial cells (PICs), myocardial infarction, cardiac repair and regeneration

## Abstract

We have previously shown that skeletal muscle-derived Sca-1^+^/PW1^+^/Pax7^−^ interstitial cells (PICs) are multi-potent and enhance endogenous repair and regeneration. Here, we investigated the regenerative potential of PICs following intramyocardial transplantation in mice subjected to an acute myocardial infarction (MI). MI was induced through the ligation of the left anterior descending coronary artery in 8-week old male C57BL/6 mice. 5 × 10^5^ eGFP-labelled PICs (MI + PICs; n = 7) or PBS (MI-PBS; n = 7) were injected intramyocardially into the border zone. Sham mice (n = 8) were not subjected to MI, or the transplantation of PICs or PBS. BrdU was administered via osmotic mini-pump for 14 days. Echocardiography was performed prior to surgery (baseline), and 1-, 3- and 6-weeks post-MI and PICs transplantation. Mice were sacrificed at 6 weeks post-MI + PICs transplantation, and heart sections were analysed for fibrosis, hypertrophy, engraftment, proliferation, and differentiation of PICs. A significant (*p* < 0.05) improvement in ejection fraction (EF) and fractional shortening was observed in the MI-PICs group, compared to MI + PBS group at 6-weeks post MI + PICs transplantation. Infarct size/fibrosis of the left ventricle significantly (*p* < 0.05) decreased in the MI-PICs group (14.0 ± 2.5%), compared to the MI-PBS group (32.8 ± 2.2%). Cardiomyocyte hypertrophy in the border zone significantly (*p* < 0.05) decreased in the MI-PICs group compared to the MI-PBS group (330.0 ± 28.5 µM^2^ vs. 543.5 ± 26.6 µm^2^), as did cardiomyocyte apoptosis (0.6 ± 0.9% MI-PICs vs. 2.8 ± 0.8% MI-PBS). The number of BrdU+ cardiomyocytes was significantly (*p* < 0.05) increased in the infarct/border zone of the MI-PICs group (7.0 ± 3.3%), compared to the MI-PBS group (1.7 ± 0.5%). The proliferation index (total BrdU+ cells) was significantly increased in the MI-PICs group compared to the MI-PBS group (27.0 ± 3.4% vs. 7.6 ± 1.0%). PICs expressed and secreted pro-survival and reparative growth factors, supporting a paracrine effect of PICs during recovery/remodeling. Skeletal muscle-derived PICs show significant reparative potential, attenuating cardiac remodelling following transplantation into the infarcted myocardium. PICs can be easily sourced from skeletal muscle and therefore show promise as a potential cell candidate for supporting the reparative and regenerative effects of cell therapies.

## 1. Introduction

Myocardial infarction (MI) is characterised by a lack of blood flow to the heart resulting in significant cell death and reduction in cardiomyocytes with pathological hypertrophy of surviving cardiomyocytes and scar formation or fibrosis [1]. Although sufferers with MI are surviving and living longer due to advances in early diagnosis and intervention, therapeutic treatment to date only prevents further exacerbation of the damaged myocardium rather than encouraging cardiac repair and regeneration. Studies suggest that the level of endogenous cardiomyocyte renewal is low <1% and after MI, this modest rate of renewal is unable to replace dead cardiomyocytes [2,3]. Pre-clinical studies over the last decade have spurred on the potential of utilising stem and progenitor cells and cardiomyocytes derived from stem cells to replace the lost cells of the damaged heart after MI [4,5,6]. Most recently, cell transplantation into the MI heart has been shown to stimulate endogenous repair and regeneration due to an autocrine/paracrine mechanism [7,8,9,10]. Limited engraftment and survival of injected cells post-implantation has hindered the development of cell therapy as an effective strategy for cardiac regeneration. In recent year’s attention has turned towards in vivo cellular reprogramming and the use of biomaterials and tissue engineering to rebuild and replace cardiac tissue. Gabisonia et al. (2019) showed that delivery of human microRNA-199a in infarcted pig hearts can stimulate cardiac repair, through cardiomyocyte de-differentiation and proliferation, leading to marked improvements in both global and regional contractility, increased muscle mass and reduced scar size. However, subsequent persistent and uncontrolled expression of the microRNA resulted in myocardial infiltration of proliferating cells displaying a poorly differentiated myoblastic phenotype and sudden arrhythmic death of most of the treated pigs [11]. Therefore, as with cell therapy, dosage and duration of treatment needs to be optimised in order to develop safe and effective cardiac repair and regeneration strategies.

In the developing heart, Sca-1 expression is detected at a relatively late stage (E13.5), and increases with myocardial differentiation [12]. Sca-1 is a glycosyl phosphatidylinositol anchored cell surface protein that has been widely used as a marker to isolate hematopoietic stem cells [13]. In the postnatal heart, the cardiac non-myocyte cell (NMC) fraction contains a subset of Sca-1 cells that demonstrate cardiogenic potential [14]. Interestingly, the number of Sca-1-expressing cells increases in the border zone after myocardial infarction, suggesting a role for this cell subset in cardiac repair [15]. Post-transplantation MI neonates, Sca-1 cells have the capacity to home to the heart and play a role in endogenous cardiac progenitor cell expansion and survival [16]. In the adult murine heart, Sca-1 positive CD31 negative cells show cardiomyocyte, endothelial and smooth muscle lineage potential after cardiac grafting, augmenting cardiac function [17]. These characteristics make Sca-1^+^ cells an attractive therapeutic target for heart repair and regeneration.

Skeletal muscle-derived Sca-1^+^/PW1^+^/Pax7^−^ interstitial cells (PICs) are a myogenic stem/progenitor cell population but are distinct from satellite cells due to their lack of Pax7 expression and localisation within the interstitial space [18,19]. We have previously shown that PICs exhibit properties of stem/progenitor cells being clonogenic, self-renewing, and multi-potent in vitro and in vivo [18,19]. We have demonstrated that PICs can differentiate into cells of the 3 cardiac lineages; cardiomyocyte, smooth muscle and endothelial cells [18,19]. We also showed that PICs secrete a multitude of pro-regenerative growth factors and cytokines and when injected into the damaged skeletal muscle they accelerate and improve endogenous repair and regeneration [20]. Moreover, the effect of PIC transplantation was greater than injecting IGF-1 and HGF growth factors [20]. Given the difficultly and low yield of harvesting stem/progenitor cells directly from the myocardium from a translational perspective, PICs represent a plausible cell candidate [21].

The aim of the present study was to determine the regenerative and reparative potential of PICs when transplanted into the myocardial infarcted mouse heart. 

## 2. Materials and Methods

### 2.1. Isolation of PICs from Skeletal Muscle

Cells were isolated as previously described [19]. Briefly skeletal muscle hind limb from 21-day-old mice was dissected and rinsed in basic buffer (MEM, 2.93 mM Hepes, 2.05 mM glutamine, 9.99 mM taurine, pH 7.3). Tissue was minced and stirred at 37 °C for 30 min in digestion buffer (basic buffer with 7 mg/mL collagenase II). The sample was then centrifuged at 300× *g* for 1 min and the cell supernatant retained. The cell supernatant was passed through a 40 μM filter, and topped up to 30 mL with incubation buffer (basic buffer with 0.5% bovine serum albumin (BSA), pH 7.3) and spun at 1500 rpm for 7 min after which the supernatant was discarded, and the cell pellet re-suspended in 1 mL incubation medium (PBS, 0.5% BSA, 2 μM EDTA). The cells were purified for the PIC population using magnetic-activated cell sorting (MACs) as per standard protocol (Miltenyi). Briefly, using direct mouse CD45 microbeads (Miltenyi) the CD45^+^ cells were depleted from the cell preparation, leaving the CD45^–^ fraction; from this, the Sca-1^+^ cells were then enriched using an indirect (FITC) mouse Sca-1 microbead kit (Miltenyi). 

### 2.2. Cell Culture

Cells were cultured on 1.5% gelatin coated dishes in growth medium (Supplementary Table S1). Cultures were incubated at 37 °C in 5% CO_2_ and passaged 1 in 3 when they reached ~80% confluency using 0.25% trypsin-EDTA solution (Supplementary Table S1). Single cell-derived clonal colonies were generated by serial dilution seeding of one cell per well of a 1.5% gelatin-coated 96-well tissue culture plate. The clone C9 was selected and used in this study [19]. C9 PICs (P10) were transduced for 24 h using a 1:5 ratio of lentiviral supernatant:PICs media containing 8 μg/mL polybrene, as previously described, transduction was done to introduce GFP expression in to C9 PICs for tracking engrafted cells post injection [19]. 

### 2.3. Animals

All experimental procedures were conducted in accordance with the regulations for animal testing, directed by the Home Office and stipulated under the Animals (Scientific Procedures) Act 1986. 8–10 week-old male C57BL/6 mice (Charles River, Margate, UK) were used. Mice were group housed in conditions of 22 ± 1 °C room temperature and 50 ± 1% relative humidity on a 12 h light-dark cycle. Standard laboratory mice chow and water were available ad libitum. Mice were randomly assigned to a treatment group. The groups were: MI + PICs; n = 7; MI-PBS; n = 7; and Sham n = 8. 

### 2.4. Echocardiography

Prior to echocardiography, mice were weighed and anaesthetised with 0.5 L/min O_2_ and 4% Isoflurane. Mice were placed onto an imaging station (Vevo, Amsterdam The Netherlands) where anaesthesia was regulated to maintain heart rate within 400–500 beats per minute monitored by ECG recordings. Body temperature was maintained at 37 ± 0.5 °C using a temperature probe. Following depilation, parasternal long and short axis images of the heart were taken using the Vevo 770 imaging system (The Netherlands) and measurements taken in triplicate using the Bright-mode and Motion-mode features. Ultrasounds were taken prior to inducing MI, baseline (BL), 7 days, 21 days and 42 days post-MI or Sham surgery. Ultrasounds were taken by an experienced researcher who was blinded to each treatment group. The following measurements were taken: interventricular septum thickness at end-systole/diastole (IVS_s/d_), left ventricular end diastolic volume/ left ventricular end systolic volume (LVEDV/LVESV), and left ventricular anterior wall thickness at end-systole/diastole (LVAW_s/d_). Ejection Fraction (EF), Stroke Volume (SV), and Fractional Shortening (FS) were measured manually using the left ventricular interventricular dimension (LVID) Trace tool and were also derived by long and short axis calculations. 

### 2.5. Acute Myocardial Infarction Surgery

Mice were subjected to myocardial infarction (MI) as previously described [22,23]. Briefly, mice were sedated under deep anaesthesia using 4% isoflurane and 1 L/min O_2_ confirmed by the absence of the pinch withdrawal reflex and the change in breathing pattern. Mice were intubated and attached to a mechanical ventilator (200 mL stroke volume; 150 breaths per minute, Hugo Sachs Electronik, March-Hugstetten, Germany) anaesthesia was maintained at 2% isoflurane and 1 L/min O_2_. The thoracic area was depilated, sterilised and the heart exposed by a left sided thoracotomy between the fourth and fifth rib. Following the separation of the pericardial sac, the left anterior descending (LAD) coronary artery was ligated using 6-0 silk suture (Ethicon, New Jersey, United States of America; USA) using the left atrium and aorta as entry and exit landmarks, respectively. Following a permanent ligation, the heart was assessed for atrial fibrillation for a 10 min interval. In sham-treated mice, a permanent ligature was not tied around the LAD coronary artery. Immediately following MI, mice were injected with 5 × 10^5^ GFP^+^ PICs delivered in a total volume of 15 µL PBS intramyocardially, across two sites at the border zone. Alternatively, mice were treated with PBS (Control) at a total volume of 15 µL, delivered across two sites at the border zone. The two injection sites were administered adjacent to each other along the border zone axis with 30–40 mm distance apart. 5-0 silk suture (Ethicon, New Jersey, USA) was used to close the thorax and the skin. Directly following surgery, mice were implanted with an osmotic mini pump, subcutaneously (Alzet^®^, Cupertino, CA, USA) loaded with a 0.2 M solution of BrdU (MP Biomedicals), releasing the thymidine analogue for 14 days at 0.5 µL/h. Anaesthesia was then removed and mice were taken off mechanical ventilation after observing independent breathing dyssynchronous to that of the ventilator. Methadone analgesic was administered, Comfortan^®^ (1 mg/kg; Dechra) i.m., and mice were kept in heat boxes overnight in a constant warm environment (27 ± 1 °C and a relative humidity of 50 ± 5%). 24- and 48 h following surgery, body weight and signs of pain were assessed, and post analgesic was administered as and when necessary. Following surgery, mice were singly caged and housed with access to water and chow ad libitum. Twenty-four hours post-surgery, one mouse from the MI-PBS and one mouse from the MI-PICs group were sacrificed by schedule 1 due to poor recovery.

To confirm the consistency of the infarct size across mice, the investigator performing the surgery validated their reproducibility of performing the LAD coronary ligation to lead to 40–50% damage to the LV. A sub-set of mice (n = 5) were subjected to ligation of the left coronary artery followed by the systemic administration of 1% Evans blue. Hearts were harvested and sectioned into 750 µM thick sections from the base to the apex. Images were acquired and the ischemic area/total LV was measured.

### 2.6. Tissue Harvesting, Processing and Sectioning

Mice were sacrificed by cervical dislocation at week 6 post-MI and PIC transplantation. A laparotomy was performed and diaphragm removed to expose the heart. The heart was arrested in diastole with an intra cardiac dose of cadmium chloride solution (Sigma-Aldrich, Missouri, USA) 0.1 mL of 0.3 mg/mL. The right atrium was incised, and the heart perfused through the apex with 0.1 M PBS followed by 10% formalin (Sigma-Aldrich, Missouri, USA). Hearts were excised and immersed into a 10% formalin solution for 24 h at room temperature (RT) followed by a 70% ethanol solution for 24 h. Hearts were cut into 3 parts; apex, mid and base (Supplementary Figure S1) and processed in a tissue processor (TP1020, Leica, Milton Keynes, UK). Following 18 h, hearts were embedded in wax using the modular tissue embedding system (Leica, Milton Keynes, UK).

The paraffin-embedded tissue blocks were sectioned at 6µm thick using a microtome (Reichert-jung, 1140/autocut), with the sample side facing outward to the blade. Ribbon sections were treated with ethanol to remove creases and placed in a 45 °C water bath (Raymond A Lamb). The ribbon was separated into single sections and mounted on to Superfrost Plus slides (Thermo Scientific, Waltham, MA, USA).

### 2.7. Immunohistochemistry

Sections were de-waxed at 60 °C for 15 min, cleared with xylene (Sigma-Aldrich, St. Louis, MO, USA) for 5 min and dehydrated in 100% ethanol solution 3 times for 3 min each. Sections were rehydrated using distilled water (dH_2_O). 

Antigen retrieval was performed by treating sections with Citric Acid buffer for 10 min at 100 °C. Following washes in PBS, sections were blocked with donkey serum (1:10 dilution Sigma, D9663) at RT for 1 h. Sections were immunostained using antibodies against BrdU, GFP, cleaved caspase-3 (CC3), wheat germ agglutinin (WGA), α-sarcomeric actin, calponin and vWF. The details of the primary and secondary antibodies, the dilutions used, and incubation times are shown in Supplementary Table S2. The buffer used for washes was PBS. DAPI (Sigma-Aldrich, MO, USA) was used to visualise nuclei and was applied to sections at 1 mg/mL for 5 min at RT. Sections were washed in PBS and mounted on to coverslips using Vectashield mounting medium (Vector Laboratories, Newark, CA, USA). For co-staining with GFP, α-sarcomeric actin, calponin and vWF sections were incubated with filtered 0.2% *w*/*v* Sudan Black B (Sigma-Aldrich, MO, USA) for 10 min at RT, following the DAPI step. 

Sections were visualised with a Zeiss, Axioplan 2 imaging fluorescent microscope with Axiovision software for capturing the images or a point scanning inverted confocal microscope (Nikkon, Tokyo, Japan, model 3.71 s). Twenty fields of view (FOV) in the risk region (RR) and non-risk region (non-RR) were captured per mouse and saved as tiff files for offline analysis. For cardiomyocyte hypertrophy, 100 cardiomyocytes per mouse within the sub-endocardium border zone layer were measured using ImageJ software assessing both the cross-sectional area and the circumference of each cardiomyocyte. The number of BrdU^+^ cardiomyocyte nuclei or CC3^+^ cardiomyocyte nuclei were counted in the border and infarct zone for each mouse and expressed as a percent of total cardiomyocyte nuclei per FOV. The number of BrdU^+^ cells or GFP^+^ cells that expressed α-sarcomeric actin, calponin or vWF were counted in the border and infarct zone per mouse, and expressed as a percentage of total DAPI nuclei per FOV. 

### 2.8. Hematoxylin VAN Gieson Staining for Fibrosis

Sections were deparaffinised as above and rinsed in running tap water for 1 min to wash and rehydrate the tissue. Slides were dipped into haematoxylin stain (Clin-tech, Gills haemalum, Guildford, UK) for 5 min. Excess stain was rinsed under running dH_2_O and slides were placed into Van Gieson solution (Fisher, Concord, NH, USA) for 5 min at RT. Sections were then rinsed with dH_2_O and left to dry at 60 °C for 1 h. Sections were dehydrated with 100% ethanol, followed by clearing with xylene solution twice for 5 min. DPX Mountant (Sigma-Aldrich, MO, USA) was used to mount coverslips onto slides that were left to set at RT for 1 h in the fume hood. Sections were visualised under the light microscope (Zeiss, Axioskop, Leica, Milton Keynes, UK) connected to Axiovision software where whole sections were captured at x5 magnification. Images were analysed using ImageJ software to quantify fibrosis as a percentage of the whole left ventricle.

### 2.9. Real-Time Quantitative Polymerase Chain Reaction (RT–qPCR)

Total mRNA was obtained from PICs pellets using the QIAshredders and RNeasy mini kit (Qiagen, Hilden, Germany). Final mRNA concentration (ng/μL) and quality (260/280 and 260/230 ratio) of the resulting flow though was measured using a Nanodrop 2000. cDNA was synthesised via reverse transcription, using a Taqman reverse transcription kit (Life Technologies, Carlsbad, CA, USA). qRT-PCR was performed on a Biorad CFX-connect Real-Time PCR System with a MyIQ detection system, using IQ SYBR Green supermix, 1 μL of template cDNA, and 500 nM of forward and reverse primers with the following program:

1. 95 °C—5 min
2. 40 cycles of:
  a. 95 °C—15 s
  b. 60 °C—30 s
  c. 72 °C—30 s

Primers sequences are listed in Supplementary Table S3. Florescence was detected as the end of each amplification cycle (step 2c). Melt curve analysis was performed on all reactions at 0.5 °C increments between 55 °C and 95 °C to detect any genomic DNA contamination, primer dimers, and/or non-specific amplification. Data were analysed using BioRad IQ software, and the transcript copy number estimated by normalising results to the housekeeping genes (HKG) GAPDH, ß-actin, and B2M. All reactions were run in triplicate.

### 2.10. Preparation of PIC Conditioned Media

PICs at passage five were seeded at a density of 10,000 cells/cm^2^. At 80% confluence, cells were washed in PBS three times and media were replaced with serum-free DMEM. After 72 h, the media was collected and centrifuged (Eppendorf, Hauppauge, NY, USA) at 300× *g* for 5 min. The supernatant was filtered through a 0.22 μm filter (Pall Corporation, Port Washington, NY, USA) and stored at −80 °C until required for ELISA or angiogenesis assay.

### 2.11. ELISA

The levels of mouse IGF-1 (R&D), VEGF (R&D), HGF (R&D), follistatin (Aviva Biosystems) and TGFß1 (R&D) were measured using ELISA, following the manufacturer’s instructions. Briefly, ELISA plates were incubated with standards, conditioned media from PICs or non-conditioned media (control) in triplicate for 2 h at room temperature, wells were rinsed with wash buffer five times and incubated with conjugate antibody or biotinylated antibody in the case of follistatin for 2 h at room temperature. After the second wash, follistatin plates only were incubated with 1x Avidin-HRP conjugate for 1 h at 37 °C before all plates were incubated with substrate solution for 20–30 min at room temperature or 37 °C in the case of follistatin. The addition of stop solution concluded the reaction and a microplate reader (Dynex Technologies, Chantilly, USA) was used to detect the signals at 450 nm with correction at 570 nm.

### 2.12. Angiogenesis Assay

Tube formation was assessed using The Millipore^®^ in vitro angiogenesis assay kit (Millipore, Burlington, MA, USA). The Matrigel (ECMatrix^®^) and diluent buffer was thawed overnight at 4 °C. 50 µL of a 1:10 dilution of ECMatrix^®^ was seeded into individual wells in a 96 well plate (Thermo, Waltham, MA, USA). Following a two-hour incubation at 37 °C, HUVECs at passage two were seeded at 5 × 10^3^ cells/well and were incubated in either; HUVEC media (positive control), serum-free DMEM (negative control) or PICs conditioned serum free DMEM (PIC conditioned media) for 15 h at 37 °C. Each condition was repeated in triplicate and images of 3 FOV per well were acquired with a Nikkon Eclipse Ti (Tokyo, Japan) at ×20 magnification. Image J (National Institutes of Health, Bethseda, MA, USA) was used to quantify the total number of branch points and the total capillary tube length.

### 2.13. Statistical Analysis

Data are Mean ± SEM or Mean ± SD for BrdU+ cardiomyocytes and CC3+ cardiomyocytes. Echocardiography data was analysed blind using a two-way ANOVA and Tukey’s multiple comparison tests to compare the mean between each group at each time-point. One-way ANOVA with Tukey’s multiple comparison tests were used for the hypertrophy, proliferation cell index, CC3+ cardiomyocytes, BrdU+ cardiomyocytes, fibrotic area and angiogenesis data. All analyses and measurements were carried out blind. All analyses were done using GraphPad Prism 8 (GraphPad Software, Inc., La Jolla, CA, USA). *p* < 0.05 was considered as statistically significant.

## 3. Results

### 3.1. Improvement in Cardiac Function after PICs Transplantation in MI Mice 

**Figure 1:**
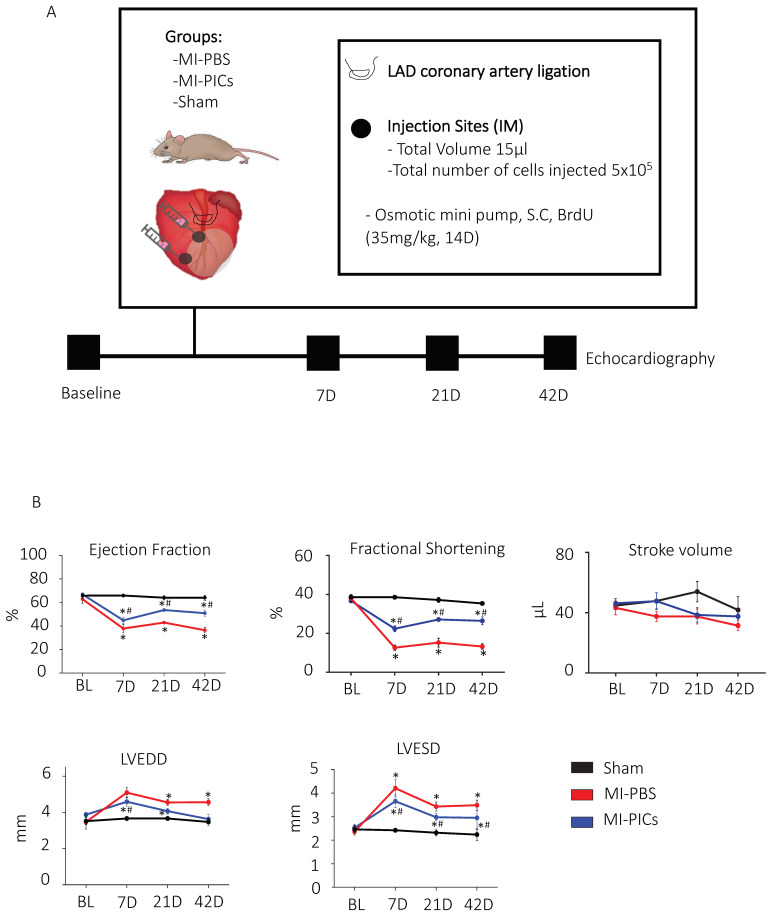
PIC transplantation improves cardiac function after MI. (**A**) Schematic diagram of experimental design and echocardiography measurements were taken, before myocardial infarction (MI) (baseline); 7 days, 21 days and 42 days after MI. Post BL echocardiography, animals underwent MI surgery with PBS, MI with PICs or Sham surgery. (**B**) Changes in cardiac function post-MI. EF = ejection fraction, FS = fractional shortening, SV = stroke volume, LVEED = left ventricular end diastolic dimension, LVESD = left ventricular end systolic dimension. Sham = 8, MI-PICS = 7 and MI-PBS = 7. Data expressed as Mean ± SEM, * *p* < 0.05 vs. Sham, # *p* < 0.05 vs. MI-PBS.

The experimental design is depicted in Figure 1A. Prior to surgery, all animals underwent baseline echocardiography measurements for Ejection fraction (EF), fractional shortening (FS), stroke volume (SV), left ventricular end diastolic dimension (LVEDD), left ventricular end systolic dimension (LVESV), Interventricular septum (IVS), left ventricle inner dimension (LVID), left ventricle posterior wall (LVPW), and left ventricle anterior wall (LVAW) (Figure 1B, Supplementary Tables S4 and S5). Mice (n = 7) were injected with 500,000 GFP tagged PICs into 2 sites of the border/infarct zone directly after ligation of the LAD coronary artery. A set of mice (n = 7) did not receive PICs but PBS. Another set of mice were sham controls (n = 8). Osmotic mini-pumps were implanted to infuse BrdU over 14 days in order to track proliferation in hearts post-MI. One week following surgery, the MI-PBS and MI-PICs group showed significantly (*p* < 0.05) decreased EF compared to sham (26.7 ± 2.6% and 44.8 ± 2.6% vs. 69.3 ± 1.2%, respectively), however, the MI-PICs group showed significantly (*p* < 0.05) less impairment compared to the MI-PBS group. A similar statistically significant (*p* < 0.05) trend was found for FS (12.6 ± 1.3% and 22.3 ± 1.4% vs. 38.6 ± 0.9%, respectively), LVEDD (5.4 ± 0.2mm and 4.8 ± 0.3mm vs. 3.9 ± 0.2 mm, respectively) and LVESD (4.7 ± 0.2 mm and 3.7 ± 0.1 mm vs. 2.4 ± 0.1 mm, respectively) (Figure 1B). By week 6, the MI-PICs group showed a sustained improvement (*p* < 0.05) in EF compared to the MI-PBS group (51 ± 2.6% vs. 28.2 ± 2.8%). This trend (*p* < 0.05) was also seen in FS (26.4 ± 2.0% vs. 13.2 ± 1.4% and LVESD (3.4 ± 0.2 mm vs. 4.3 ± 0.3 mm). Interestingly, there was no significant difference in LVEDD in the MI-PICs group compared to the MI-PBS group at 6 weeks (Figure 1B). Throughout the duration of the experiment, SV did not significantly change between any groups at any matched time points (Figure 1B, Supplementary Tables S4 and S5). We show that the volume of blood in the LVID trace at diastole is significantly greater in the MI-PBS and MI-PICs groups compared to the sham group at week 1. However, by week 3, the volume of blood is significantly less in the MI-PICs group compared to the MI-PBS group and this persists at week 6. The volume of blood in LVID trace at systole is significantly reduced in the MI-PICs group compared to the MI-PBS group at week 1. Both groups have higher blood volumes compared to the sham group. By week 3 there is no difference in the volume of blood in the left ventricle between the sham group and the MI-PICs group whilst the MI-PBS group show a persistent high volume of blood in the LV at systole (Supplementary Table S5).

### 3.2. PIC Transplantation Attenuates Cardiac Remodelling Post-MI

**Figure 2:**
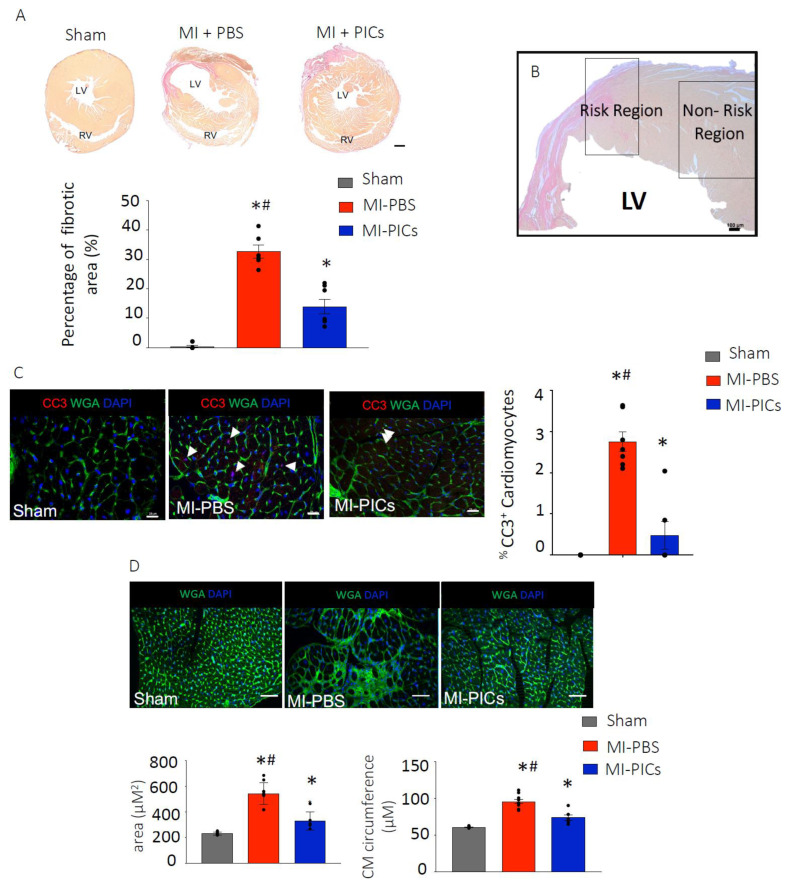
PIC transplantation attenuates cardiac remodelling after MI. (**A**) Fibrosis detection using hematoxylin van geisen (HVG) staining in Sham = 8, MI-PICS = 7 and MI-PBS = 7, pink/red staining indicates fibrosis. Data expressed as Mean ± SEM of the fibrotic area over the total area of the left ventricle (LV), * *p* < 0.05 vs. Sham, # *p* < 0.05 vs. MI-PICs, scale bar = 500 µM. (**B**) Representative micrograph depicting the risk region (RR) and non-risk region (non-RR) in the LV. Scale bar 100 µM. (**C**) % CC3^+^ apoptotic cardiomyocytes in the RR of the LV, representative image of Sham, MI-PBS and MI-PICs. Green; wheat germ agglutinin (WGA); red; cleave caspase 3 (CC3); blue; DAPI. Arrowheads show CC3+ cardiomyocytes in MI-PBS. Arrowheads show non-cardiomyocyte CC3+ cells in MI-PICs. Sham = 8, MI-PICS = 7 and MI-PBS = 6. Scale bar = 25 µM. Data are Mean ± SEM. * *p* < 0.05 vs. Sham, # *p* < 0.05 vs. MI-PICs. (**D**) Representative confocal micrographs of the RR in Sham = 8, MI-PICs = 6 and MI-PBS = 7 groups, WGA (green) and DAPI (blue). Scale bar = 500 µM. Graphs showing the cross-sectional area and circumference of cardiomyocytes in the RR of the LV. Data expressed as Mean ± SEM, * *p* < 0.05 vs. Sham, # *p* < 0.05 vs. MI-PICs.

Six weeks post-MI, the MI-PICs group showed a significantly (*p* < 0.05) reduced level of fibrosis in the left ventricle (LV) compared to the MI-PBS group (14.0 ± 2.5% vs. 32.8 ± 2.2%). Not surprisingly, the level of fibrosis remained significantly greater in both MI-PICs and MI-PBS groups, compared to the sham group (0.5 ± 0.4%) (Figure 2A). In our analyses we focused on two areas, risk region (RR), which was defined as the region that straddled 0.5 mm either side of the border zone, and the non-risk region (non-RR) of the LV (Figure 2B). A representative RR in sham hearts was determined based on the area of colour change and patterning of viable and non-viable tissue in MI injury hearts. The non-RR is defined as being remote to the infarcted area [24,25,26]. The percentage of apoptotic cardiomyocyte nuclei identified by cleaved caspase 3 (CC3^+^) staining was significantly (*p* < 0.05) greater in the MI-PBS group (2.8 ± 0.2%) compared to the MI-PICs group (0.6 ± 0.4%) and sham group (no detection of apoptotic cardiomyocytes) in the RR of the LV at 6 weeks post-MI (Figure 2C). No apoptotic cardiomyocytes were found in the non-RR (data not shown). 

In the RR of the LV, the cardiomyocyte cross-sectional area in the MI-PICs group was significantly (*p* < 0.05) decreased when compared to the MI-PBS group (330.0 ± 28.5 µM^2^ vs. 543.5 ± 26.6 µM^2^) (Figure 2D). Similarly, in the non-RR, a significant decrease (*p* < 0.05) in cardiomyocyte cross sectional area was observed in the MI-PIC group, compared to the MI-PBS group (261.6 ± 16.2 µM^2^ and 328.2 ± 8.3 µM^2^) (Supplementary Figure S2A,B). In line with the cross-sectional area results, cardiomyocyte circumference was significantly (*p* < 0.05) smaller in the MI-PICs group compared to the MI-PBS group in the RR (73.9 ± 3.7 µM vs. 97.4 ± 2.8 µM) (Figure 2D) and in the non-RR (Supplementary Figure S2A,C).

### 3.3. PICs Engraft in the Myocardial Infarcted Heart

**Figure 3:**
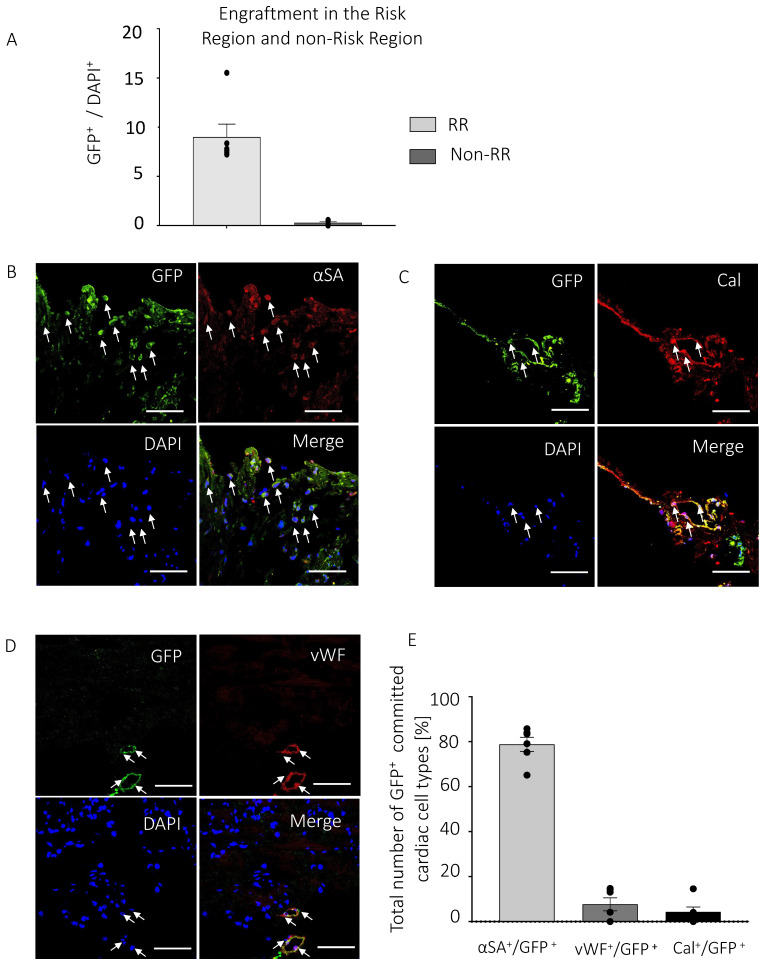
PICs engraft in the infarcted myocardium. (**A**) Percent engraftment of GFP^+^ PICs 6 weeks post-transplantation, represented as a percentage of the total number of DAPI cells in the risk region (RR) and non-risk region (non-RR). Data are Mean ± SEM in 20 FOV per mouse. (**B**–**D**) Representative confocal micrographs of GFP^+^ PICs (green) expressing markers of cardiomyocyte ((**B**), Red; α-sarcomeric actin), smooth muscle ((**C**), Red; calponin) and endothelial ((**D**), Red, vWF) cells. DAPI stain nuclei blue. Scale bar = 50 µM. (**E**) Quantification of the GFP^+^ PICs expressing markers of the cardiac tr-lineage in MI-PICs group n = 6. Data are Mean ± SEM.

Of the total number of cells in the RR, 8.9 ± 1.3% were GFP^+^ PICs at 6-weeks post-transplantation (Figure 3A). 0.2 ± 0.1% GFP^+^ PICs were detected in the non-RR. Engrafted PICs exhibited expression of proteins of the 3 main cardiac lineages (cardiomyocytes, endothelial cells and smooth muscle cells), with a large percentage (78.8 ± 3.1%) expressing α-sarcomeric actin, suggestive of commitment towards the cardiomyocyte lineage (Figure 3B,E) in the RR. However, these α-sarcomeric actin/GFP^+^ cells did not have the morphology or sarcomeric arrangement to be labelled or defined as cardiomyocytes (Figure 3B,E). To a lesser extent, 4.4 ± 1.2% of GFP^+^ PICs committed towards smooth muscle cells identified using the smooth muscle marker, Calponin (Figure 3C,E), while 7.7 ± 2.9% of GFP^+^ PICs committed towards capillaries identified using the endothelial marker, von Willebrand Factor (vWF) (Figure 3D,E). The pattern of expression of GFP together with calponin or vWF identified these cells arranged within vascular structures (Figure 3C). 

### 3.4. PICs Transplantation Increases Cell Proliferation in the MI-Heart

**Figure 4:**
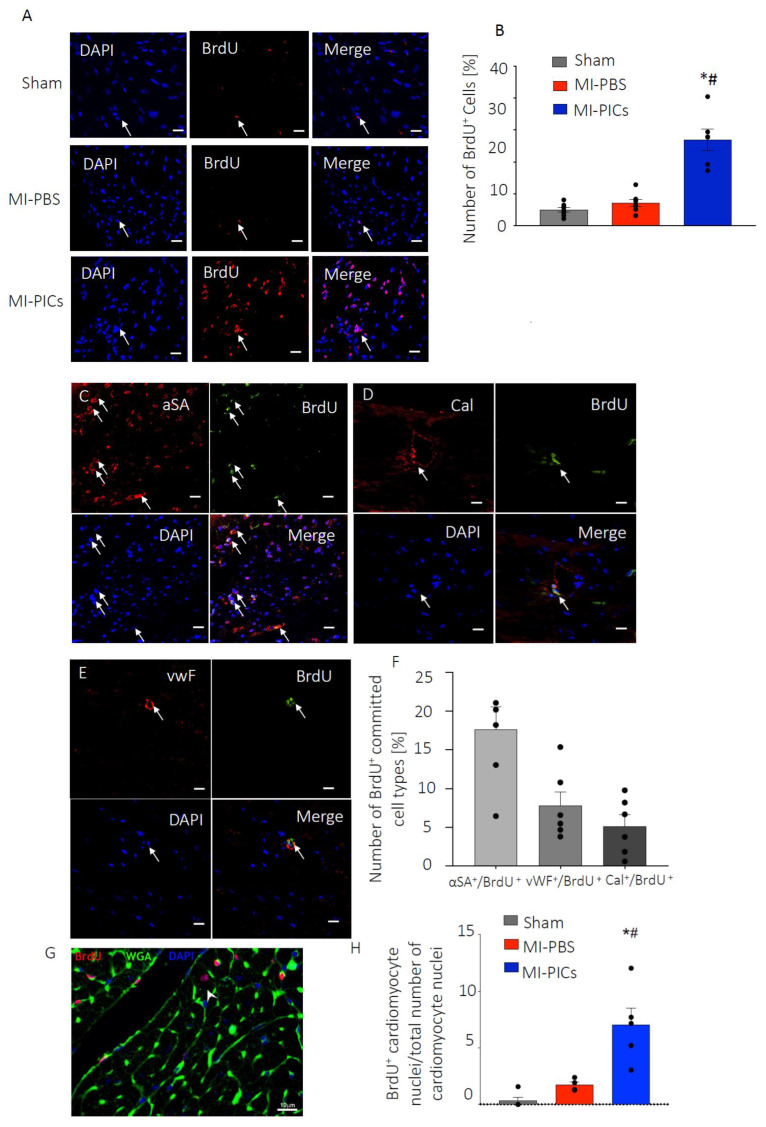
PICs transplantation increases proliferation and number of BrdU^+^ cardiomyocytes. (**A**) Representative confocal micrographs in the RR of the LV of BrdU (Red) positive cells. DAPI stain nuclei blue. Scale bar = 10 µM. Arrows indicate positive staining colocalisation. (**B**) Quantification of the number of proliferating BrdU-positive cells in the RR in Sham = 8, MI-PBS = 7 and MI-PICs = 6 groups. Data are Mean ± SEM of the total number of BrdU^+^ cells over the total number of cells (DAPI), * *p* < 0.05 vs. sham, # *p* < 0.05 vs. MI-PBS. (**C**–**E**) Representative confocal micrographs in the RR of the LV of BrdU (Green) positive, α-sarcomeric actin-expressing ((**C**), Red), calponin-expressing ((**D**), Red) or vWF-expressing ((**E**), Red) cells. DAPI stain nuclei blue. Scale bar = 10 µM; arrows indicate positive staining colocalisation. (**F**) Quantification of the number of BrdU+ trilineage cardiac expressing cells. Data are Mean ± SEM. (**G**) Representative image of BrdU^+^ cardiomyocyte (WGA) in MI-PICs. DAPI stain nuclei blue. Arrows indicate cells that show colocalisation. Sham = 5, MI-PICS = 5 and MI-PBS = 5. Scale bar = 10 µM. (**H**) Percent number of BrdU^+^ cardiomyocytes in the RR. Data are Mean ± SEM. * *p* < 0.05 vs. sham, # *p* < 0.05 vs. MI-PBS.

6-weeks after the transplantation of PICs, the MI-PICs group showed a significantly (*p* < 0.05) elevated cell proliferation (BrdU^+^) index (27.0 ± 3.4%) compared to the MI-PBS (7.2 ± 1.0%) and Sham (5.1 ± 0.9%) groups. The MI-PBS group did not show a significantly (*p* < 0.05) elevated number of proliferating cells, compared to the Sham group (Figure 4A,B). Likewise, in the non-RR, the MI-PICs group (7.1 ± 0.5%) showed a significant increase (*p* < 0.05) in cell proliferation, compared to the MI-PBS group and Sham groups (3.8 ± 0.6% vs. 3.1 ± 0.5%) (Supplementary Figure S3A,B). Proliferating BrdU^+^ cells in the RR showed increased expression of markers of all three cardiac lineages (cardiomyocytes, endothelial cells and smooth muscle cells), demonstrating their commitment to cardiac cell types (Figure 4C–F). As with the engrafted PICs, the pattern of expression of BrdU^+^/α-sarcomeric actin^+^ cells were not organised nor showed morphology of being cardiomyocytes (Figure 4C). However, the BrdU^+^ cells that expressed calponin or vWF were arranged within vascular structures (Figure 4D,E). No proliferating BrdU^+^ cardiac lineage expressing cells were observed in the non-RR of the LV. 

Finally, using WGA staining combined with BrdU and DAPI, we analysed the sections for BrdU^+^ cardiomyocyte nuclei (Figure 4G). We found a significant increase in the number of BrdU^+^ cardiomyocyte nuclei (7.0 ± 1.5%), in the MI-PICs group, compared to MI-PBS (1.7 ± 0.3%), and Sham groups (0.3 ± 0.3%) (Figure 4H). 

### 3.5. PICs Secrete an Array of Pro-Survival and Reparative Paracrine Factors

**Figure 5:**
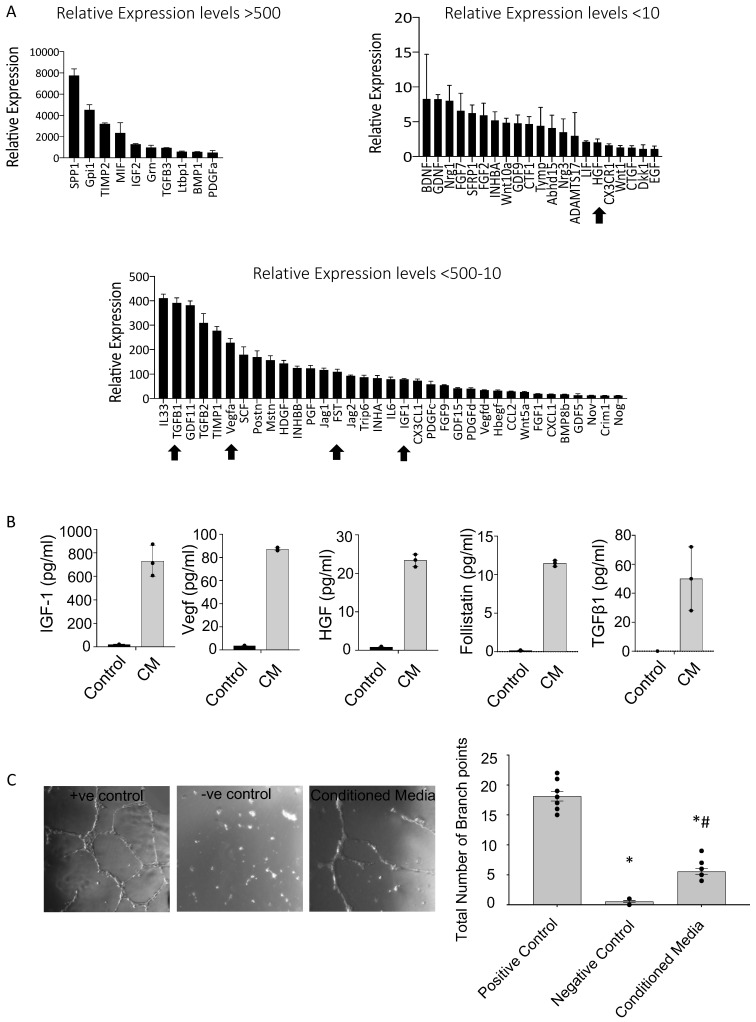
PICs have a pro-reparative secretome. (**A**) Pro-survival and regenerative transcript expression of PICs. Data are relative expression to housekeeping genes GAPDH, ß-actin and B2M. Gene profile relative expression levels are depicted as, >500, 500–10 and <10. Data are Mean ± SD, n = 3. The arrows indicate the genes that were further investigated at the protein level. (**B**) Protein quantification using ELISA; concentration of mouse IGF-1, VEGF, HGF, Follistatin and TGFß1 (pg/mL) in mPIC conditioned media (CM) relative to unconditioned (control) media. Data are Mean ± SD, n = 3. (**C**) Matrigel tube formation assay. HUVECs were cultured for 15 h in 96-well plates coated with matrigel. Data are presented as the Mean ± SEM. Positive control, HUVEC media, Negative Control, DMEM and Conditioned media; is serum free DMEM conditioned with PICs media * *p* < 0.05 vs. Positive control, # *p* < 0.05 vs. Negative control.

To identify the potential mechanism through which PICs attenuate cardiac remodeling we looked to their secretome. Measured by qRT-PCR, PICs were found to express, relative to housekeeping genes, a range of cardio-protective and pro-regenerative growth factors and cytokines measured by qRT-PCR where fold expression is in relation to housekeeping genes (Figure 5A). Pro-angiogenic factors, including vascular endothelial growth factor A (VEGFA) were relatively expressed >100 fold. Many members of the transforming growth factor beta (TGFß) superfamily and associated proteins including TGFβ1 and the TGFB superfamily antagonist, follistatin (FST) were also expressed at high levels (Figure 5A). The growth factors, insulin-like growth factor-1 (IGF-1) and hepatocyte growth factor (HGF) were also highly expressed (Figure 5A). We confirmed secreted expression at the protein level of these selected factors in PIC conditioned culture media, which supported our qRT-PCR data (Figure 5B). To support a mechanistic underpinning of PIC transplantation we performed a functional in vitro angiogenesis assay using HUVECs and the PIC conditioned culture media. Results showed that HUVECs exposed to PICs conditioned media significantly increased the total number of branch points (5.6 ± 0.4), compared to HUVECs exposed to serum-free DMEM (negative control; 0.6 ± 0.2) (Figure 5C). Not surprisingly, both of these conditions had significantly less branch points compared to the HUVEC media positive control (18.1 ± 0.5). A similar trend was observed when quantifying the total capillary tube length (Supplementary Figure S4).

## 4. Discussion

The main findings that emanate from this study are that skeletal muscle-derived Sca-1+/PW1+ PICs transplanted into the myocardially infarcted mouse heart engraft and significantly improve cardiac function and remodelling. PICs do not significantly differentiate into cardiomyocytes, and therefore did not directly contribute to the significant improvement in cardiac function and remodelling. Our findings suggest that PIC transplantation acts through an indirect paracrine mechanism of action, stimulating endogenous repair processes, including angiogenesis. 

### 4.1. Improvement in Cardiac Function after PICs Transplantation in MI Mice

Our results support the hypothesis that like other stem/progenitor cells, skeletal muscle derived Sca-1+ PICs have beneficial effects in improving cardiac function and reducing cardiac remodelling after transplantation intramyocardially in mice post-MI. We show that following acute MI, as early as 1 week post-transplantation, mice showed a higher EF compared to MI mice with no treatment. This remains persistent, showing 23% higher EF 6 weeks after treatment compared to mice with MI and no treatment. A similar observation was found with FS. Of note, MI mice treated with PICs did not recapitulate full recovery remaining significantly impaired compared to shams. Our data shows a similar trend to the intramyocardial transplantation of cardiac-derived Sca1^+^ side population (SP) cells in MI mice [17]. After 12 weeks, Sca1^+^ SP cells improved EF significantly compared to mice with MI with no treatment, however, like our results, EF remained significantly impaired compared to shams [17]. Furthermore, a study that transplanted 2 × 10^5^ Sca-1^+^ cardiac progenitor cells in MI mice significantly improved EF compared to MI mice without treatment [27], also corroborating our findings. The importance of using Sca-1^+^ stem/progenitors was elegantly demonstrated by Wang et al. (2006), where post-MI, mice transplanted with cardiac-derived Sca-1^+^/CD31^-^ cells showed significant cardiac functional improvement compared to mice treated with cardiac-derived Sca-1^-^/CD31^-^ cells, emphasising the importance of Sca-1^+^ cells for cardiac reparative therapies [15]. This was further supported in Sca-1 knock-out mice, that presented a reduced EF by 10% compared to wild type that declined further by 13% after transaortic constriction [28]; suggesting an integral role of Sca-1 in cardiac function [15]. Our findings support a Sca-1^+^ population of stem/progenitor cells isolated from skeletal muscle that has beneficial effects in improving cardiac function in mice post-MI. This cell population is easier to harvest and less invasive than cardiac-derived Sca-1^+^ stem/progenitor populations, supporting their application in the clinical setting.

### 4.2. Improved Cardiac Remodelling after PICs Transplantation in MI Mice

Delineating the heart into the RR, defined as ‘the region that straddles 0.5 mm either side of the border zone’ and the non-risk region [25], we found significant attenuated cardiac remodeling, evidenced through decreased fibrosis/infarct size, cardiomyocyte hypertrophy and apoptosis with PIC treatment, compared to no treatment. We also observed this trend in the non-RR however, the levels were lower than that found in the RR. Our findings are supported by studies that have transplanted cardiac derived Sca1^+^ cells post-MI and show decreased fibrosis, cardiomyocyte hypertrophy and apoptosis [17,28,29]. Moreover, PICs have shown a similar effect in vivo in the porcine model of skeletal muscle injury [20]. 

### 4.3. Increased Proliferation of Cardiac Cells after PICs Transplantation in MI Mice

The attenuated cardiac remodeling with PIC transplantation coincided with increased proliferation. PIC transplantation increased the number of proliferating BrdU^+^ cells, of which a proportion (~30%) were positive for cardiomyocyte (α-sarcomeric actin), smooth muscle (calponin) and endothelial (vWF) cell markers. The BrdU^+^ cells that were also positive for smooth muscle and endothelial markers were organised within vascular structures, indicative of newly formed blood vessels following PIC transplantation. PIC transplantation also increased the number of BrdU^+^ cardiomyocytes (~6%), suggesting cardiomyocyte proliferation following PIC transplantation. Increased number of new, proliferating vasculature cells and cardiomyocytes in the infarct/border zone following stem/progenitor cell transplantation is in agreement with other studies [30,31,32]. When comparing our findings to other studies there is disparity in the proliferative rates observed between studies. One study has reported a low proliferation index in the presence of adult cardiac-derived Sca1^+^ cells in mice subjected to cardiac pressure overload [5,28], whilst others have reported significantly higher rates [15,16]. These discrepancies between studies, including ours, could be due to Sca 1^+^ cells being purified and transplanted as a mixed population, without the rigor taken to purify for a single cell progeny, and as was carried out here [17]. Other factors potentially affecting the proliferation index are differences in the number of cells transplanted and delivery route of administration. The Sca-1^+^ PIC population used in our study are clonogenic, being derived from a single Sca-1^+^ PIC, and self-renewing [19]. Moreover, PICs therapeutic effects have been validated in other models of skeletal muscle injury and in larger animal models [18,19,20]. The BrdU+ cells in both the RR and non-RR that did not express cardiac lineage markers could represent a number of different cell types, such as fibroblasts or immune cells, activated post-MI and PIC transplantation. These proliferating cells play an important role in cardiac remodelling, support, and extracellular vesicles communication to regulate cardiac function [32]. During development, cardiac fibroblasts stimulate cardiomyocyte proliferation [33]. Immune cells play an integral part in both repair by scar formation and the initiation of tissue regeneration and it has been shown that macrophages directly contribute collagen to the forming post-injury scar [34]. The significance and role of the different proliferating non-cardiac cell types in cardiac repair and regeneration strategies warrants further investigation.

### 4.4. Transplanted PICs Engraft but Do Not Significantly Differentiate into Mature Cardiomyocytes

The present study showed that transplanted PICs engrafted in the infarcted myocardium and some differentiated into smooth muscle and endothelial vascular cells, which were also arranged within vascular structures. However, the present data failed to show that transplanted PICs differentiated into mature functional cardiomyocytes, at least after 6 weeks post-transplantation. A large majority of the engrafted PICs expressed the protein α-sarcomeric actin, suggestive of being committed towards the cardiomyocyte lineage [35]. However, these cells did not have the morphology or organised sarcomeric protein expression to be classified as cardiomyocytes. In agreement with previous studies, cardiac-derived Sca1^+^ cells have been shown to have the ability to differentiate to the cardiac tri-lineage post-transplantation in the MI heart [17,36,37]. However, although studies suggest a significant and lasting contribution of Sca-1^+^ cells to cardiomyocytes in normal physiological ageing [17,38], the transplanted PICs in our study did not fully differentiate into mature cardiomyocytes, and therefore would not have directly contributed to the significant improvement in cardiac function that we found with PIC transplantation. Uchida et al. (2013) suggest that there are abundant types of Sca-1^+^ cells in the heart, and that only a small subset of Sca-1^+^ cells can truly differentiate into cardiomyocytes [37]. As the PICs in the present study were derived from skeletal muscle this could explain their lack of true differentiation potential into the cardiomyocyte cell type [37]. 

There were a low proportion of engrafted PICs (~9.1% of PICs, accounting for ~0.8% of the total number of cells) that did not commit to the cardiac tri-lineage. Due to the multi-potency of PICs, these cells could have differentiated into another lineage and in particular skeletal muscle. However, these GFP+ cells remained small and rounded and did not morphologically appear as multi-nucleated myotubes. Moreover, even if transplanted PICs did differentiate into skeletal muscle myofibers the functional data suggests the number of these differentiated cells was not significant enough to have an effect on function, i.e., we did not record any ventricular arrhythmias. Previous studies in both animal models and human clinical trials have shown the development of ventricular arrhythmias following skeletal muscle myoblast transplantation in the MI heart [39]. Further investigation into the importance of the fraction of remaining non-cardiac GFP+ engrafted PICs is warranted. Due to the limited cardiomyocyte differentiation potential of the engrafted PICs, but together with improved cardiac function, remodelling and proliferation index in the present study, we postulated that the mechanism of action of PICs was that of a paracrine provider and activator of endogenous repair processes [7,9]. Over the past decade there has been a paradigm shift of the mechanism of action of adult stem cell transplantation in cardiac regenerative therapy from that of differentiation of transplanted cells into mature cell types to the transplanted cells acting through a paracrine mechanism to activate endogenous repair processes [7] leading to improved cardiac function and remodelling [40,41,42,43,44,45]. 

### 4.5. PICs Express and Secrete Pro-Survival and Regenerative Factors That Stimulate Angiogenesis

Our transcript and protein analysis revealed that PICs expressed and secreted a multitude of pro-survival and pro-regenerative paracrine factors, such as IGF-1, VEGFA and HGF. It has been previously shown that IGF-1 improved cardiomyocyte survival [43,45,46], protected cardiomyocytes from hypertrophic and oxidative stress [47,48], which reduced pathological cardiac remodelling and improved ventricular function [49,50,51]. When overexpressed in MSCs, IGF-1 combined with HGF enhanced neovascularisation with a moderate improvement in cardiac regeneration after administration in a porcine model of MI [48]. HGF alone combined with MSCs attenuated ischemic myocardial fibrosis [49], suggesting the importance of these growth factors expressed by PICs in cardiac repair and regeneration.

The growth factor VEGFA has been shown to stimulate the recruitment of endothelial cells and initiated vascularisation following injury [52,53,54]. Increased VEGFA expression and secretion from PICs in the present study could explain the increased BrdU^+^ endothelial cells within vascular structures following PICs transplantation post-MI. Indeed, we showed in vitro that PICs conditioned media stimulated angiogenesis supporting the in vivo data. We also detected increased levels of members of the transforming growth factor (TGF)-β family in PICs. A large body of evidence suggests that this family of growth factors critically regulates the inflammatory and reparative response following infarction [55,56,57]. 

### 4.6. Limitations

In this study, we have used a murine model of MI that can cause varying degrees of damage to the left ventricle. To minimise this an experienced researcher with over a decade of experience performing LAD coronary artery ligation, performed all of the surgeries for this study. To further avoid bias, all data was analysed blind by a different researcher. Another consideration in this study is that an acute model of MI and cell treatment was investigated. The efficacy of PICs transplantation may be more beneficial using a chronic model, where treatment of PICs can be delivered once pathological manifestations have established. Additionally, to be more representative of MI in the human heart the therapeutic potential of PICs should be investigated in an ischemic/reperfusion model of MI, which has more consistent levels of left ventricular damage [58,59]. Moreover, the use of the cardiac ischemic/reperfusion model would have more translatability to the clinical arena. The present study did not address whether route of administration or number of doses would affect the outcome. Previous studies have shown that cardiac progenitor cells injected systemically through the tail vein, home and engraft to the infarcted heart [10,35].

This study focused on a 6-week follow up post-cell transplantation into the heart and although we found that a high proportion of PICs expressed α-sarcomeric actin, we would require a longer follow-up to determine if these cells had matured into functional cardiomyocytes or another cell type, such as skeletal muscle. The present study did not ascertain whether the BrdU^+^ cells present in the post-MI heart were the transplanted PICs or endogenous cells (i.e., fibroblasts or macrophages). As the transplanted GFP^+^ PICs expressed cardiomyocyte (α-sarcomeric actin; 78.8%), smooth muscle (calponin; 4.4%) and endothelial (vWF; 7.7%) cell lineage markers (Figure 3) and a proportion of the BrdU^+^ cells were committed towards the cardiomyocyte (α-sarcomeric actin; 17.7%), smooth muscle (calponin; 5.1%) and endothelial (vWF; 7.7%) cell lineage (Figure 4), it is argued that a portion of the BrdU^+^ cells were the transplanted PICs. That the PICs are a self-renewing and clonogenic population [19] would support their proliferative potential post-transplantation. A longer follow-up would also be necessary to determine the significance of the potentially undifferentiated PICs and whether they remain engrafted in the heart for an extended period of time post-transplantation or their uncontrolled proliferation. We have previously shown that the PICs used in this study do not form tumours when tested in a tumour formation assay in vivo [19], and we did not see any tumour formation when PICs were injected intramyocardially in this study. Finally, based on our in vitro findings we have postulated that the beneficial effects of PICs are through a paracrine mechanism, however further experiments are needed to determine whether PICs secrete the same pro-survival and pro-regenerative factors in vivo and actively cross-talk with endogenous cells.

## 5. Conclusions

We demonstrate that PICs transplanted into the myocardial infarcted mouse heart improve cardiac function, and attenuate cardiac remodeling by reducing fibrosis, cardiomyocyte hypertrophy, apoptosis and increasing cell proliferation and angiogenesis. Transplanted PICs differentiated into smooth muscle and endothelial cells, which were organized within vascular structures, but not cardiomyocytes. Given their limited differentiation capacity into cardiomyocytes, as has been shown with different Sca1^+^ cell populations mentioned previously, the main mechanism of action of PICs is likely that of a paracrine effect. Indeed, PICs expressed and secreted an array of pro-survival and pro-reparative factors, which have a beneficial stimulatory effect on endogenous cardiac repair post-MI [9,60,61,62] and stimulated angiogenesis in vitro in this study. The findings of this study support PICs as a potential therapeutic cell candidate for cardiac repair. PICs can be easily sourced from skeletal muscle, and can undergo in vitro propagation with the prospect of generating large numbers of these cells to be used in cellular reparative therapies [18,19]. The present study provides important insight into the use of PICs as a progenitor cell therapy for the treatment of MI. 

## Data Availability

The datasets used and/or analysed during the current study are available from the corresponding author on reasonable request.

## Supplementary Materials

The following supporting information can be downloaded at: https://www.mdpi.com/2073-4409/11/24/4050/s1, Figure S1: Cardiac landmarks; Figure S2: Hypertrophic cardiomyocytes in the non-RR, Figure S3: Proliferation index in the non-RR, Figure S4: Total capillary tube tube formation length Table S1: Cell culture maintenance and passage media; Table S2: List of primary and secondary antibodies, Table S3: List of primers, Table S4: Echocardiography parameters, Table S5: Echocardiography trace parameters
