# Correction: Ruchaya et al. Transplantation of Skeletal Muscle-Derived Sca-1^+^/PW1^+^/Pax7^−^ Interstitial Cells (PICs) Improves Cardiac Function and Attenuates Remodeling in Mice Subjected to Myocardial Infarction. *Cells* **2022**, *11*, 4050

**DOI:** 10.3390/cells13110895

**Published:** 2024-05-23

**Authors:** Prashant J. Ruchaya, Fiona C. Lewis-McDougall, Nitiphat Sornkarn, Sachin Amin, Benjamin Grimsdell, Abeer Shaalan, Guilia Gritti, Kyi Thar Soe, James E. Clark, Georgina M. Ellison-Hughes

**Affiliations:** 1 Centre for Human and Applied Physiological Sciences, School of Basic and Medical Biosciences, Faculty of Life Sciences & Medicine, King’s College London, Guy’s Campus, London SE1 1UL, UK; f.lewis@qmul.ac.uk (F.C.L.-M.); nitiphat.sornkarn@kcl.ac.uk (N.S.); sachin.amin@kcl.ac.uk (S.A.); benjamin.grimsdell@kcl.ac.uk (B.G.); shaalan.abeer@kcl.ac.uk (A.S.); g.gritti@kcl.ac.uk (G.G.); 2 Centre for Gene Therapy and Regenerative Medicine, School of Basic and Medical Biosciences, Faculty of Life Sciences & Medicine, King’s College London, Guy’s Campus, London SE1 1UL, UK; 3 School of Health, Sport and Biosciences, Stratford Campus, University of East London, London E16 2RD, UK; u1819492@uel.ac.uk; 4 The William Harvey Research Institute, Charterhouse Square, Barts & The London School of Medicine & Dentistry, Queen Mary University of London, London EC1M 6BQ, UK; 5 Rayne Institute, School of Cardiovascular and Metabolic Medicine and Sciences, Faculty of Life Sciences & Medicine, King’s College London, St Thomas’ Campus, London SE1 7EH, UK; james.2.clark@kcl.ac.uk

In the original publication [1], there were mistakes in Figure 1A, Figure 2A,C,D, Figure 3A,D, Figure 4B,F,H, and Figure 5B,C as they were published.

In Figure 1A, the syringes depicting the location of transplanted cells should have been transparent. In Figure 2A, the image of the sham heart was cropped out and could not be visualized completely. In Figure 2C, the panels Sham and MI-PBS were the same, and the graphs were missing all colors. In Figure 2D, the graphs were missing all colors. In Figure 3A,E, Figure 4B,F,H, and Figure 5B,C, the graphs were also missing all colors.

The corrected Figure 1A, Figure 2A,C,D, Figure 3A,D, Figure 4B,F,H, and Figure 5B,C appear below. The authors state that the scientific conclusions are unaffected. This correction was approved by the Academic Editor. The original publication has also been updated.

**Figure 1:**
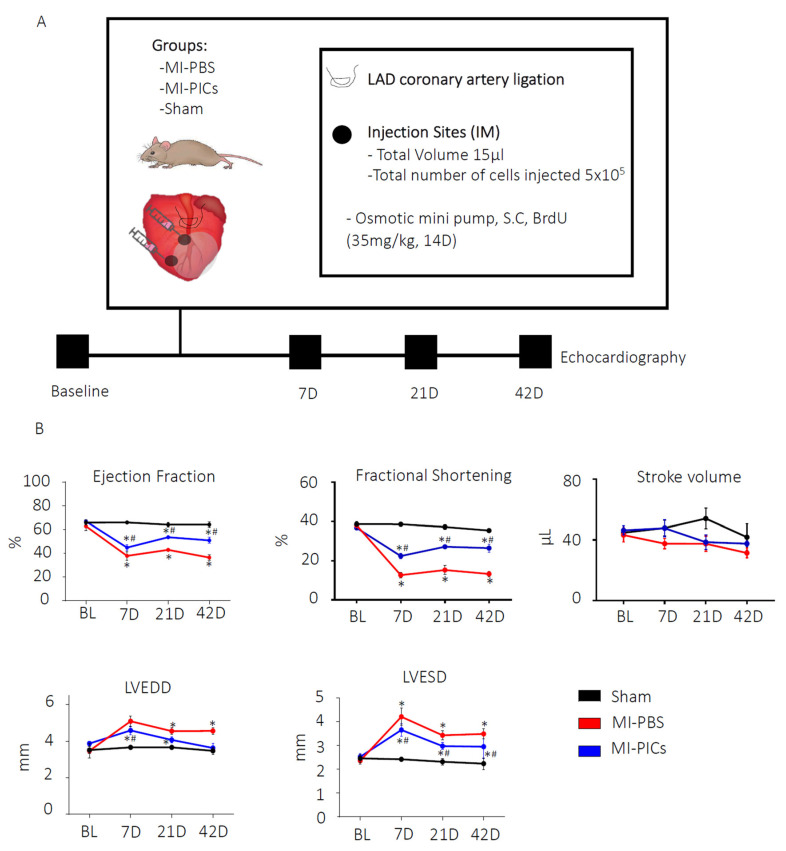
PIC transplantation improves cardiac function after MI. (**A**) Schematic diagram of experimental design and echocardiography measurements were taken, before myocardial infarction (MI) (baseline); 7 days, 21 days and 42 days after MI. Post BL echocardiography, animals underwent MI surgery with PBS, MI with PICs or Sham surgery. (**B**) Changes in cardiac function post-MI. EF = ejection fraction, FS = fractional shortening, SV = stroke volume, LVEED = left ventricular end diastolic dimension, LVESD = left ventricular end systolic dimension. Sham = 8, MI-PICS = 7 and MI-PBS = 7. Data expressed as Mean ± SEM, * *p* < 0.05 vs. Sham, # *p* < 0.05 vs. MI-PBS.

**Figure 2:**
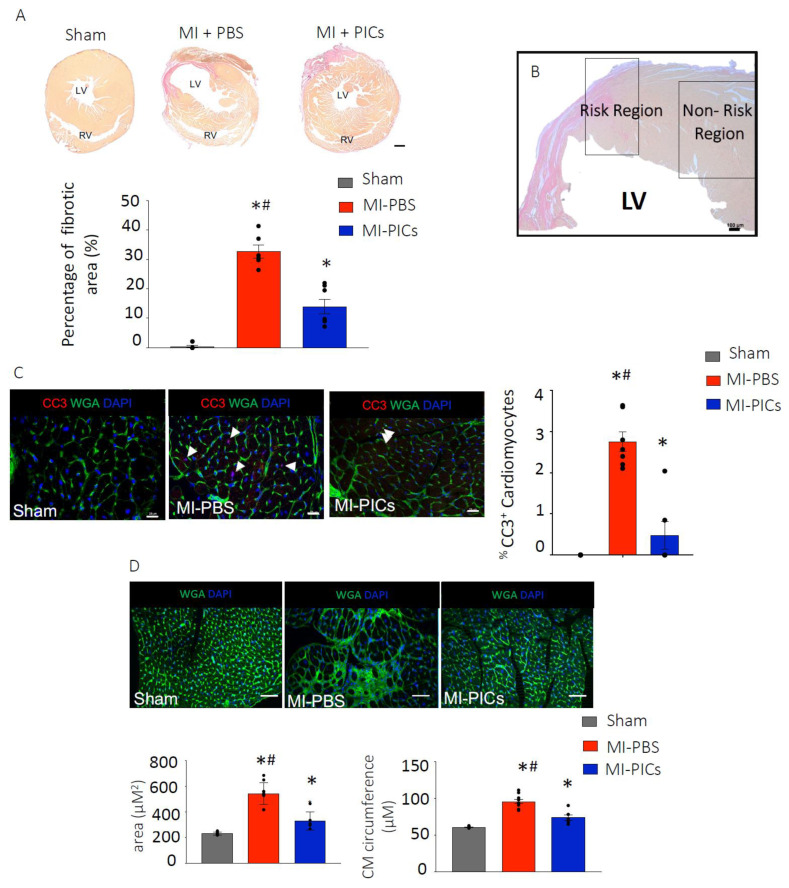
PIC transplantation attenuates cardiac remodelling after MI. (**A**) Fibrosis detection using hematoxylin van geisen (HVG) staining in Sham = 8, MI-PICS = 7 and MI-PBS = 7, pink/red staining indicates fibrosis. Data expressed as Mean ± SEM of the fibrotic area over the total area of the left ventricle (LV), * *p* < 0.05 vs. Sham, # *p* < 0.05 vs. MI-PICs, scale bar = 500 µM. (**B**) Representative micrograph depicting the risk region (RR) and non-risk region (non-RR) in the LV. Scale bar 100 µM. (**C**) % CC3+ apoptotic cardiomyocytes in the RR of the LV, representative image of Sham, MI-PBS and MI-PICs. Green; wheat germ agglutinin (WGA); red; cleave caspase 3 (CC3); blue; DAPI. Arrowheads show CC3+ cardiomyocytes in MI-PBS. Arrowheads show non-cardiomyocyte CC3+ cells in MI-PICs. Sham = 8, MI-PICS = 7 and MI-PBS = 6. Scale bar = 25 µM. Data are Mean ± SEM. * *p* < 0.05 vs. Sham, # *p* < 0.05 vs. MI-PICs. (**D**) Representative confocal micrographs of the RR in Sham = 8, MI-PICs = 6 and MI-PBS = 7 groups, WGA (green) and DAPI (blue). Scale bar = 500 µM. Graphs showing the cross-sectional area and circumference of cardiomyocytes in the RR of the LV. Data expressed as Mean ± SEM, * *p* < 0.05 vs. Sham, # *p* < 0.05 vs. MI-PICs.

**Figure 3:**
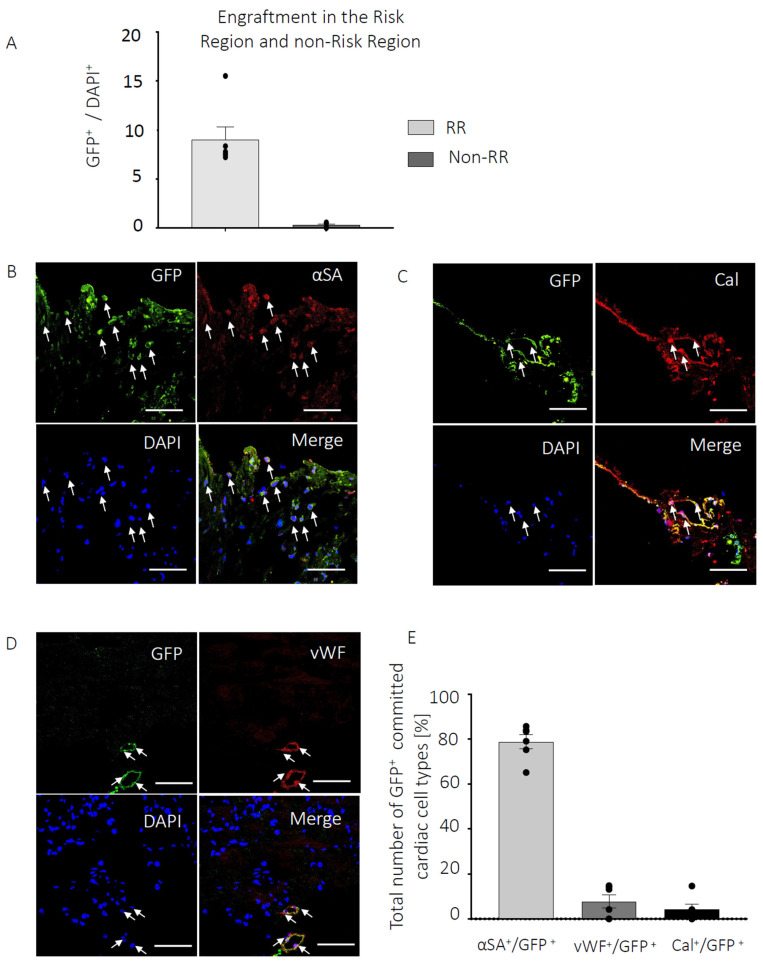
PICs engraft in the infarcted myocardium. (**A**) Percent engraftment of GFP+ PICs 6 weeks post-transplantation, represented as a percentage of the total number of DAPI cells in the risk region (RR) and non-risk region (non-RR). Data are Mean ± SEM in 20 FOV per mouse. (**B**–**D**) Representative confocal micrographs of GFP+ PICs (green) expressing markers of cardiomyocyte ((**B**), Red; α-sarcomeric actin), smooth muscle ((**C**), Red; calponin) and endothelial ((**D**), Red, vWF) cells. DAPI stain nuclei blue. Scale bar = 50 µM. (**E**) Quantification of the GFP+ PICs expressing markers of the cardiac tr-lineage in MI-PICs group n = 6. Data are Mean ± SEM.

**Figure 4:**
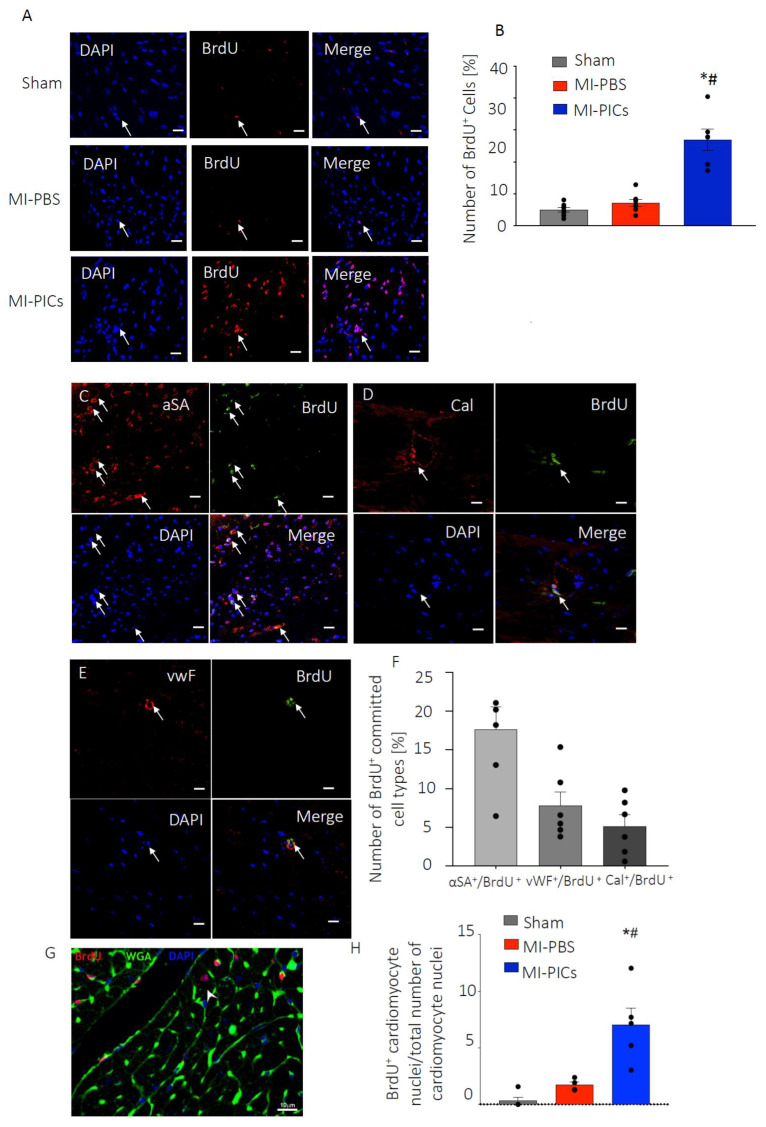
PICs transplantation increases proliferation and number of BrdU^+^ cardiomyocytes. (**A**) Representative confocal micrographs in the RR of the LV of BrdU (Red) positive cells. DAPI stain nuclei blue. Scale bar = 10 µM. Arrows indicate positive staining colocalisation. (**B**) Quantification of the number of proliferating BrdU-positive cells in the RR in Sham = 8, MI-PBS = 7 and MI-PICs = 6 groups. Data are Mean ± SEM of the total number of BrdU^+^ cells over the total number of cells (DAPI), * *p* < 0.05 vs. sham, # *p* < 0.05 vs. MI-PBS. (**C**–**E**) Representative confocal micrographs in the RR of the LV of BrdU (Green) positive, α-sarcomeric actin-expressing ((**C**), Red), calponin-expressing ((**D**), Red) or vWF-expressing ((**E**), Red) cells. DAPI stain nuclei blue. Scale bar = 10 µM; arrows indicate positive staining colocalisation. (**F**) Quantification of the number of BrdU^+^ trilineage cardiac expressing cells. Data are Mean ± SEM. (**G**) Representative image of BrdU^+^ cardiomyocyte (WGA) in MI-PICs. DAPI stain nuclei blue. Arrows indicate cells that show colocalisation. Sham = 5, MI-PICS = 5 and MI-PBS = 5. Scale bar = 10 µM. (**H**) Percent number of BrdU^+^ cardiomyocytes in the RR. Data are Mean ± SEM. * *p* < 0.05 vs. sham, # *p* < 0.05 vs. MI-PBS.

**Figure 5:**
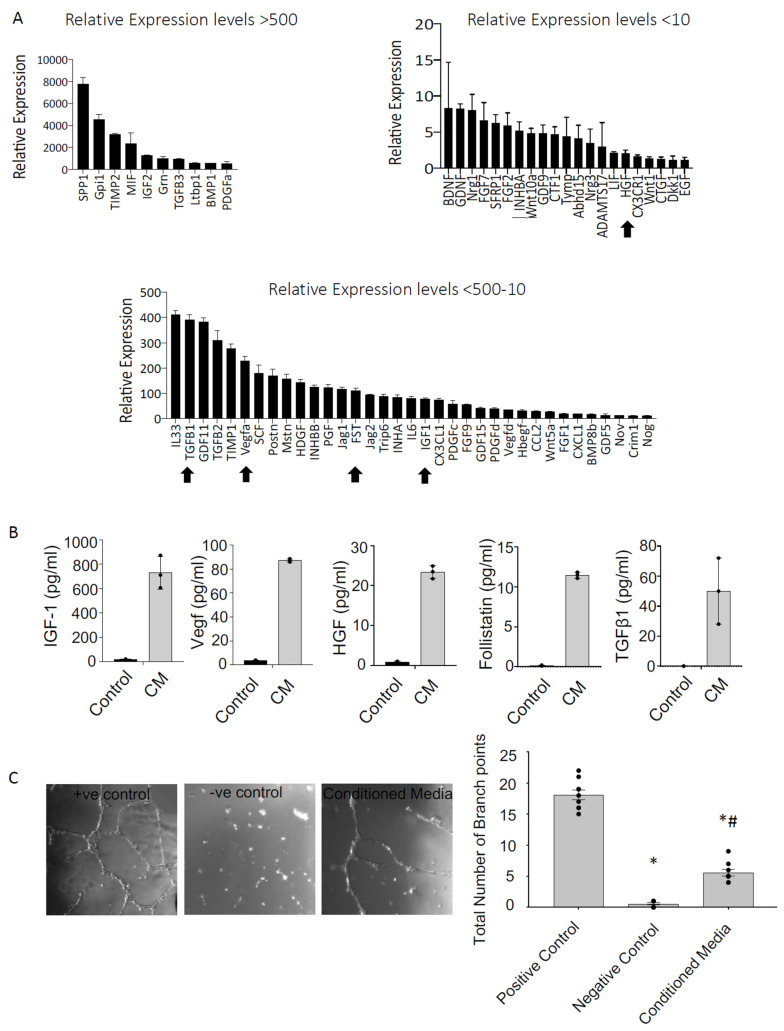
PICs have a pro-reparative secretome. (**A**) Pro-survival and regenerative transcript expression of PICs. Data are relative expression to housekeeping genes GAPDH, ß-actin and B2M. Gene profile relative expression levels are depicted as, >500, 500–10 and <10. Data are Mean ± SD, n = 3. The arrows indicate the genes that were further investigated at the protein level. (**B**) Protein quantification using ELISA; concentration of mouse IGF-1, VEGF, HGF, Follistatin and TGFß1 (pg/mL) in mPIC conditioned media (CM) relative to unconditioned (control) media. Data are Mean ± SD, n = 3. (**C**) Matrigel tube formation assay. HUVECs were cultured for 15 h in 96-well plates coated with matrigel. Data are presented as the Mean ± SEM. Positive control, HUVEC media, Negative Control, DMEM and Conditioned media; is serum free DMEM conditioned with PICs media * *p* < 0.05 vs. Positive control, # *p* < 0.05 vs. Negative control.
