# Supplementary material for: Transplantation of Skeletal Muscle-Derived Sca-1+/PW1+/Pax7− Interstitial Cells (PICs) Improves Cardiac Function and Attenuates Remodeling in Mice Subjected to Myocardial Infarction
Source: Cells. 2022 Dec 12;11(24):4050. doi: 10.3390/cells11244050 (PMC9776789; doi:10.3390/cells11244050)
Supplement: Supplementary file 1 [file cells-11-04050-s001.zip › cells-1975842-supplementary.pdf]

Media	Components
**Growth Media**	100ml DMEM/F12 (Sigma), 1ml ITS (Life Technologies), 100ml Neurobasal Medium (Life Technologies), 1ml Glutamax (Life Technologies), 2ml B27 (Life Technologies), 1ml N2 (Life Technologies), 44µl EGF (Peprotech), 22µl bFGF (Peprotech), 22ml ESCQ-FBS (Life Technologies), 10ng/ml LIF (Millipore) 1ml PenStrep (Life Technologies), 100µl Fungizone (Life Technologies), 100µl Gentamicin (Sigma)
**Passage Media**	177.6ml DMEM/F12 (Sigma), 20ml ESCQ-FBS, 2ml PenStrep (Life Technologies), 200µl Gentamicin (Sigma), 200µl Fungizone (Life Technologies)

Supplementary Table S1. Cell culture maintenance and passage media

Antibody	Source	Dilution	Incubation time	Temperature
**Primary antibody**				
anti-BrdU	Abcam ab1893	1:500	1 hour	RT
anti-WGA	Thermo Fisher W11261	1:500	1 hour	RT
anti-GFP	Rockland 32778	1:500	1 hour	RT
Anti-actin-α-sarcomere	Sigma A2172	1:1000	1 hour	RT
Anti-calponin	Abcam ab46794	1:500	1 hour	RT
Anti-vWF	Abcam ab7356	1:500	1 hour	RT
Anti-CC3	Cell Signalling asp165	1:200	1 hour	RT
**Secondary Antibody**				
Alexa Fluor 633 donkey anti-goat	Life Technologies a21082	1:500	1 hour	RT
Alexa Fluor 594donkey anti-goat	Life Technologies a11058	1:500	1 hour	RT
Alexa Fluor 488 donkey anti-goat	Life Technologies a11055	1:500	1 hour	RT
Alexa Fluor 594 donkey anti-mouse	Life Technologies a21203	1:500	1 hour	RT
Alexa Fluor 594 donkey anti-rabbit	Life Technologies r37119	1:500	1 hour	RT
Alexa Fluor 647 donkey anti-rabbit	Life Technologies a31573	1:500	1 hour	RT

Supplementary Table S2. List of primary and secondary antibodies

Official Full Name	Alias	Forward Primer	Reverse Primer	Accession Number
Abhydrolase Domain Containing 15	Abhd15	TCCTACTTGGGGGAGTGTGG	GGCATACCTGCTGAGAGAGAT	NM_026185.4
ADAMTS16	ADAMTS16	GGTCCTTTGCACCCTAGCAT	CAGGTGTCTGCTGCTCACTG	NM_172053.3
ADAMTS17	ADAMTS17	GCGAGTCCCTGCTCTATCAC	CCGACAGCTGTGCCCG	NM_001033877.4
Brain-derived neurotrophic factor	BDNF	ATCCACTGAGCAAAGCCGAA	CCTGGTGGAACATTGTGGCT	NM_007540.4
Bone morphogenetic protein 1	BMP1	TACAGCCAGGGCAGGAGTAT	CTTGCTGAGTCGGGTCCTTT	NM_009755.3
Bone morphogenetic protein 2	BMP2	ACTGCGGTCTCCTAAAGGTCG	ACCTGGGGAAGCAGCAACAC	NM_007553.3
Bone morphogenetic protein 4	BMP4	GCAACCCAGCCTGAGTATCT	ATGGCTCCATTGGTTCCTGC	NM_007554.3
Bone morphogenetic protein 6	BMP6	GGCTCCGGTTCTTCAGACTA	TGGCATTCATGTGTGCGTTG	NM_007556.3
Bone morphogenetic protein 8b	BMP8b	TGATGATGGCCACGGTTCTC	AATGACAGAGTCCAGCCAGC	NM_007559.5
Bone morphogenetic protein 10	BMP10	AGCTTCAAGAACGAAGATCTGTTT	TTTTACGGTCCACGCCATCA	NM_009756.3
Crim1	Crim1	GGATCGAAGAAGAGAAGCCAGA	GGTAAAGGACAGCACTCCCC	NM_015800.3
Connective tissue growth factor	Ctgf	AGAACTGTGTACGGAGCGTG	GTGCACCATCTTTGGCAGTG	NM_010217.2
CCL2	CCL2	AGCTGTAGTTTTTGTCACCAAGC	GTGCTGAAGACCTTAGGGCA	NM_011333.3
Cardiotrophin1	CTF1	AGAGGGAGGGAAGTCTGGAAGA	TTGGCCTCCAAATGGGGTAG	NM_007795.1
chemokine (C-X-C motif) ligand 1	CXCL1	ACCGAAGTCATAGCCACACTC	CTCCGTTACTTGGGGACACC	NM_008176.3
chemokine (C-X-C motif) ligand 9	CXCL9	TGTGGAGTTCGAGGAACCCT	AGTCCGGATCTAGGCAGGTT	NM_008599.4
chemokine (C-X3-C motif) ligand 1	CX3CL1	GCGACAAGATGACCTCACGA	TGTCGTCTCCAGGACAATGG	NM_009142.3
CX3CR1	CX3CR1	CCATCTGCTCAGGACCTCAC	CACCAGACCGAACGTGAAGA	NM_009987.4

Supplementary Table S3. List of primers used

Official Full Name	Alias	Forward Primer	Reverse Primer	Accession Number
dickkopf WNT signaling pathway inhibitor 1	Dkk1	GACAACTACCAGCCCTACCC	GATCTGTACACCTCCGACGC	NM_010051.3
Epidermal growth factor	EGF	AGGATCCTGACCCCGAACTT	ACAGCCGTGATTCTGAGTGG	NM_010113.4
Acidic fibroblast growth factor	FGF1	TTATACGGCTCGCAGACACC	TCTCCGCATGCTTCTTGGAG	NM_010197.3
Fibroblast growth factor 2 (basic)	FGF2	GGCTGCTGGCTTCTAAGTGT	GTCCCGTTTTGGATCCGAGT	NM_008006.2
Fibroblast growth factor 7	FGF7	GAGAGGCTCAAGTTGCACGA	CGGTTGCTCCTTGACTTTTGT	NM_008008.4
Fibroblast growth factor 9	FGF9	GCAGTCACGGACTTGGATCA	AATTCCAGAATGCCGAAGCG	NM_013518.4
Fibroblast growth factor 23	FGF23	GGCACTGCTAGAGCCTATCC	TCAGGGCACTGTAGATGGTC	NM_022657.4
Follistatin	Fst	CTGAGAAAGGCCACCTGCTT	TCTTCACAGGACTTTGCTTTGAT	NM_001301373.1
Growth differentiation factor 2	GDF2	GCTTCAGCGTGGAAGATGCTA	GGCAGGAGACATAGAGTCGG	NM_019506.4
Growth differentiation factor 5	GDF5	AAAGGGCAAGATGACCGAGG	TAAGATCCGCAGTTCAGCCC	NM_008109.3
Growth differentiation factor 9	GDF9	CAACCAGGTGACAGGACCG	AGCAAGTGTTCCATGGCAGT	NM_008110.2
Growth differentiation factor 10	GDF10	GCAGACATCGGGTGGAATGA	ATGGGCGGACAATCTTGGG	NM_145741.3
Growth differentiation factor 11	GDF11	GAGGGGCTGCATCCTTTCAT	GGAGCAGTAGTTGGCCTTGT	NM_010272.2
Growth differentiation factor 15	GDF15	TCCAGAGGTGAGATTGGGGT	GCTTCAGGGGCCTAGTGATG	NM_011819.3
Glial cell-derived neurotrophic factor	GDNF	ACCAGTGACTCCAATATGCCTG	GCTGCCGCTTGTTTATCTGG	NM_010275.3

Supplementary Table S3 continued. List of primers used

Official Full Name	Alias	Forward Primer	Reverse Primer	Accession Number
Granulin	Grn	CTGCCCGTTCTCTAAGGGTG	ATCCCCACGAACCATCAACC	NM_008175.4
Glucose-6-phosphate isomerase	Gpi1	TGTCTACGAACACGGCCAAA	AGTCCAATGGCTGACCACAG	NM_008155.4
Hepatoma-derived growth factor	HDGF	AAACTTGGGCTCGCGCTTC	GGCATCTCATCAATCCGGGC	NM_008231.4
Hepatocyte growth factor-like	HGF	TGCTCCTCCCTTCCCTACTC	CCGGGCTGAAAGAATCAAAGC	NM_001289458.1
Heparin-binding EGF-like growth factor	Hbegf	GCATCCAAAGTGATCGCTGC	GACAACACTGCGGCCAGAAA	NM_010415.2
Interleukin 1, alpha	IL1a	GCTTGAGTCGGCAAAGAAATCA	AGATGGTCAATGGCAGAACTGT	NM_010554.4
Interleukin 1, beta	IL1b	TGCCACCTTTTGACAGTGATG	TGATGTGCTGCTGCGAGATT	NM_008361.4
Interleukin 6	IL6	CTCTGCAAGAGACTTCCATCCA	GACAGGTCTGTTGGGAGTGG	NM_031168.2
Interleukin 10	IL10	GGCGCTGTCATCGATTTCTC	ATGGCCTTGTAGACACCTTGG	NM_010548.2
Interleukin-33	IL33	GGCTCACTGCAGGAAAGTACA	TTGGTCTTCTGTTGGGATCTTCT	NM_001164724.2
Insulin-like growth factor 1	IGF1	TCTGCCTCTGTGACTTCTTGA	TAGCCTGTGGGCTTGTTGAA	NM_010512.5
Insulin-like growth factor 2	IGF2	AAAAGCCATCTCCCCGTTCT	GGATCCCCATTGGTACCTGG	NM_010514.3
Inhibin, alpha	INHA	AGCAATGGATGGGGAAGGTG	GTGGCACCTGTAGCTGGG	NM_010564.5
Inhibin, beta A	INHBA	GCTGCTCAAGTGCCAATACC	GTTTTGTCAGCCGGCTCTTG	NM_008380.2
Inhibin, beta B	INHBB	TCAGCTTTGCAGAGACAGATGG	CCTTGACCCGTACCTTCCTC	NM_008381.4
Jagged 1	Jag1	AGCCAAGGTGTGCGGG	ACGCGGGACTGATACTCCTT	NM_013822.5
Jagged 2	Jag2	GGGTGGCAACTCCTTCTACC	AGCTCCTCATCTGGAGTGGT	NM_010588.2
latent TGF-beta binding protein	Ltbp1	CCCGAAGTGGTAACAGCACC	GCACAGGAGGGGATGTCTTTT	NM_019919.4
Leukemia inhibitory factor	LIF	ACAACTGTGCAGACTGTGGA	CAGTAAGGGCACAATCCCTGC	NM_008501.2
Myostatin	Mstn	GAGCCCAGGCACTGGTATTT	AGGGATTCAGCCCATCTTCTC	NM_010834.3
macrophage migration inhibitory factor	MIF	CTTTGTACCGTCCTCCGGTC	GTGCACTGCGATGTACTGTG	NM_010798.2

Supplementary Table S3 continued. List of primers used

Official Full Name	Alias	Forward Primer	Reverse Primer	Accession Number
Noggin	Nog	GAGGAGGGAAAAGGCTCGTC	CGGGCATCCGAGATTACTCC	NM_008711.2
nephroblastoma overexpressed gene	Nov	GAGTGCTGCGAAAAGTGGAC	CTATAGGCTGGAAGGGCCAAG	NM_010930.4
Neuregulin-1	Nrg1	TCATAGTGAAAGGTATGTATCAGCC	GGAGACATTTCCGAAGGGGG	NM_178591.2
Neuregulin-2	Nrg2	GCCAGATCCTAAGCAAAAGGC	TCTGCCGGAGATGATGATGC	NM_001167891.1
Neuregulin 3	Nrg3	GATCCAACAGACCACTTGGGG	AATGCTGCACAGAACATGCC	NM_008734.3
Neurotrophin 3	Ntf3	CTGATCGGATCCGGAGGAAG	CCGCAGCCACTCATCATTATC	NM_001164034.1
Periostin	Postn	CTGCTTCAGGGAGACACACC	TCTGAAGTCAGTGTGGTGGC	NM_015784.3
Platelet-derived growth factor subunit A	PDGFa	GAGGAGGAGACAGATGTGAGG	AGGAGAACAAAGACCGCACG	NM_008808.3
Platelet-derived growth factor C	PDGFc	AAGGAACAGAACGGAGTGCAAG	TGAGGAAACTTCGGGCTGTG	NM_019971.3
Platelet-derived growth factor D	PDGFd	CAACCTCAGGAGAGATGAGAGC	TTTTCTCCTGGGAACGGAGC	NM_027924.3
placental growth factor	PGF	GACGAGCATGGTGATTGTGC	AGGCACCACTTCCACTTCTG	NM_008827.3
Kit ligand	SCF	TTACCTTCGCACAGTGGCTG	TCATCCACACAAGGTCACGG	NM_013598.3
secreted phosphoprotein 1	SPP1	CTGGCAGCTCAGAGGAGAAG	CAGCATTCTGTGGCGCAAG	NM_001204201.1
Secreted frizzled related protein 1	SFRP1	CAAGCGAGTTTGCACTGAGG	AGTTGTGGCTGAGGTTGTCC	NM_013834.3
TIMP metallopeptidase inhibitor 1	TIMP1	AGAGACACACCAGAGCAGATACC	TGGTCTCGTTGATTTCTGGGG	NM_001044384.1
TIMP metallopeptidase inhibitor 2	TIMP2	CAAAGCAGTGAGCGAGAAGG	TGTCAGGTCCTTTGAACATCTTT	NM_011594.3
Transforming growth factor, beta 1	TGFB1	CCCGAAGCGGACTACTATGC	CATAGATGGCGTTGTTGCGG	NM_011577.2
Transforming growth factor, beta 2	TGFB2	TCCCCTCCGAAAATGCCATC	ACTCTGCCTTCACCAGATTCG	NM_009367.4
Transforming growth factor, beta 3	TGFB3	GCACTTTACAACAGCACCCG	CAGACGGCCAGTTCATTGTG	NM_009368.3

Supplementary Table S3 continued. List of primers used

Official Full Name	Alias	Forward Primer	Reverse Primer	Accession Number
thyroid hormone receptor interactor 6	Trip6	GCTGTTACAAGTGTGAGGAGTG	AGAGCTCTTGGATACGCCAG	NM_011639.3
thymidine phosphorylase	Tymp	TGAATAGACTGGAGCAATGGCAG	GCCCCAATTTGTGTGTCCTG	NM_138302.2
Vascular endothelial growth factor A	Vegfa	CTTGCAGATGTGACAAGCCAA	TCAACGGTGACGATGATGGC	NM_001025250.3
Vascular endothelial growth factor D	Vegfd	GTTGTAAGTGCTTGCCCACG	GAGTGGTCTTCTGTCCCAGG	NM_010216.2
wingless-type MMTV integration site family, member 1	Wnt1	GCAACCACAGTCGTCAGAAC	GTTCACGATGCCCCACCATC	NM_021279.4
wingless-type MMTV integration site family, member 3A	Wnt3a	TGGCTCCTCTCGGATACCTC	AGCCAAGGACCACCAGATCG	NM_009522.2
wingless-type MMTV integration site family, member 5A	Wnt5a	CGCTTCGCTTGAATTCCTCG	CAATGGGCTTCTTCATGGCG	NM_009524.4
wingless-type MMTV integration site family, member 7A	Wnt7a	GGGACTATGACCCGGAAAGC	CGAAGAGAAGCCACCGATCC	NM_009527.3
wingless-type MMTV integration site family, member 10A	Wnt10a	AGATCGCCATCCATGAGTGC	ACTCTCTCGAAAACCTCGGC	NM_009518.2
Actin, beta	B-Actin	GATCAAGATCATTGCTCCTCCTG	ACGCAGCTCAGTAACAGTCC	NM_007393.5
Beta-2-microglobulin	B2M	GGCTCACACTGAATTCACCC	TGTCTCGATCCCAGTAGACGG	NM_009735.3
Glyceraldehyde-3-phosphate dehydrogenase	GAPDH	GAGAGTGTTTCCTCGTCCCG	ATGAAGGGGTCGTTGATGGC	NM_001289726.1

Supplementary Table S3 continued. List of primers used

		Sham n=8	MI-PBS n=7	MI-PICs n=7
IVS,d (mm)	Baseline	0.85±0.05	0.72±0.01	0.76±0.04
	Week 1	0.90±0.05	0.51±0.04*	0.58±0.02*
	Week 3	0.88±0.10	0.50±0.07*	0.67±0.04*
	Week 6	0.95±0.06	0.44±0.03*	0.57±0.08*
IVS,s (mm)	Baseline	1.31 ±0.11	1.35 ±0.18	1.15±0.05
	Week 1	1.35 ±0.12	0.74 ±0.09*	0.77 ±0.03*
	Week 3	1.40 ±0.08	0.68 ±0.11*	0.88 ±0.09*
	Week 6	1.30 ±0.08	0.66 ±0.06*	0.80 ±0.13*
LVID,d (mm)	Baseline	3.75 ±0.09	3.70 ±0.12	3.87±0.13
	Week 1	3.88 ±0.14	5.32 ±0.22*	4.59 ±0.24*
	Week 3	4.18 ±0.19	4.99 ±0.31*	4.07 ±0.17*
	Week 6	3.95 ±0.17	5.02 ±0.34*	3.63 ±0.25#
LVID,s (mm)	Baseline	2.42 ±0.07	2.23 ±0.15	2.53 ±0.15
	Week 1	2.56 ±0.12	4.71 ±0.21*	3.65 ±0.28*
	Week 3	2.54 ±0.10	4.39 ±0.36*	3.46 ±0.10*
	Week 6	2.55 ±0.12	4.44 ±0.31*	3.49 ±0.31*#

Supplementary Table S4. Echocardiography parameters measured at baseline, weeks 1, 3 and 6. Data expressed as means ± sem *<0.05 vs. sham, #<0.05 vs MI-PBS. Interventricular septum; IVS, left ventricle inner dimension; LVID at diastole; d and systole; s

		Sham n=8	MI-PBS n=7	MI-PICs n=7
LVPW,d (mm)	Baseline	0.95 ±0.10	0.76 ±0.03	0.62 ±0.04
	Week 1	0.85 ±0.05	0.68 ±0.12	0.61 ±0.08
	Week 3	0.78 ±0.05	0.64 ±0.06	0.67 ±0.07
	Week 6	0.91 ±0.03	0.74 ±0.13	0.82 ±0.26
LVPW,s (mm)	Baseline	1.30 ±0.12	1.30 ±0.19	1.05 ±0.06
	Week 1	1.17 ±0.08	0.94 ±0.18	0.86 ±0.10
	Week 3	1.18 ±0.05	0.89 ±0.13	0.84 ±0.06
	Week 6	1.35 ±0.08	0.93 ±0.16*	0.86 ±0.31*#
LVAW,d (mm)	Baseline	0.81 ±0.03	0.80 ±0.04	0.78 ±0.05
	Week 1	0.83 ±0.04	0.61 ±0.09*	0.59 ±0.03*
	Week 3	0.82 ±0.05	0.67 ±0.06*	0.66 ±0.06*
	Week 6	0.85 ±0.06	0.44 ±0.03*	0.59 ±0.15*
LVAW,s (mm)	Baseline	1.15 ±0.04	1.20 ±0.05	1.21 ±0.05
	Week 1	1.20 ±0.06	0.72 ±0.05*	0.91 ±0.09*
	Week 3	1.34 ±0.13	0.87 ±0.08*	0.91 ±0.07*
	Week 6	1.07 ±0.07	0.71 ±0.05*	0.89 ±0.17
LVID trace, d (μl)	Baseline	64.7±3.1	56.3±5.0	69.4±5.3
	Week 1	69.1±7.5	143.8±10.4*	109.1±14.0*
	Week 3	74.6±8.1	127.8±22.9*	71.7±8.4#
	Week 6	67.8±9.5	118.0±17.1*	64.3±2.9#
LVID trace, s (μl)	Baseline	19.9±1.4	24.7±8.1	23.2±2.5
	Week 1	21.2±2.3	106.2±9.6*	61.4±9.5*#
	Week 3	29.2±4.1	90.3±20.1*	33.1±3.8#
	Week 6	25.9±1.8	86.6±15.3*	31.3.3±2.9#

Supplementary Table S5. Echocardiography parameters measured at baseline, weeks 1, 3 and 6. Data expressed as means ± sem *<0.05 vs. sham, #<0.05 vs MI-PBS. left ventricle posterior wall; LVPW, left ventricle anterior wall; LVAW, left ventricular internal dimension trace; LVID trace at diastole; d and systole; s.

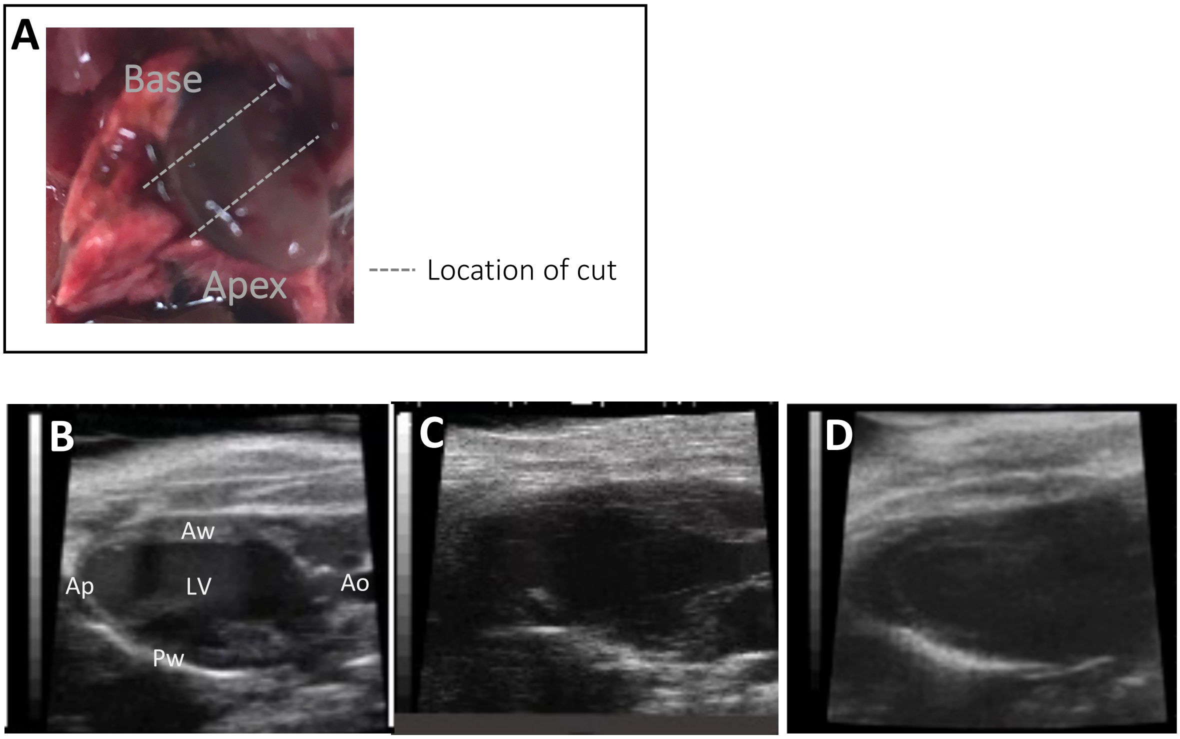

S.F.1- Photomicrograph depicting the areas of the heart dissected for ex vivo analysis. Echocardiographic Parasternal long axis images of the left ventricle in sham (B), MI-PBS (C) and MI-PICs (D), Anterior wall; (Aw), Posterior wall; (Pw), Apex; (Ap), Aorta; (Ao) and left ventricle (LV).

Supplementary figure 1

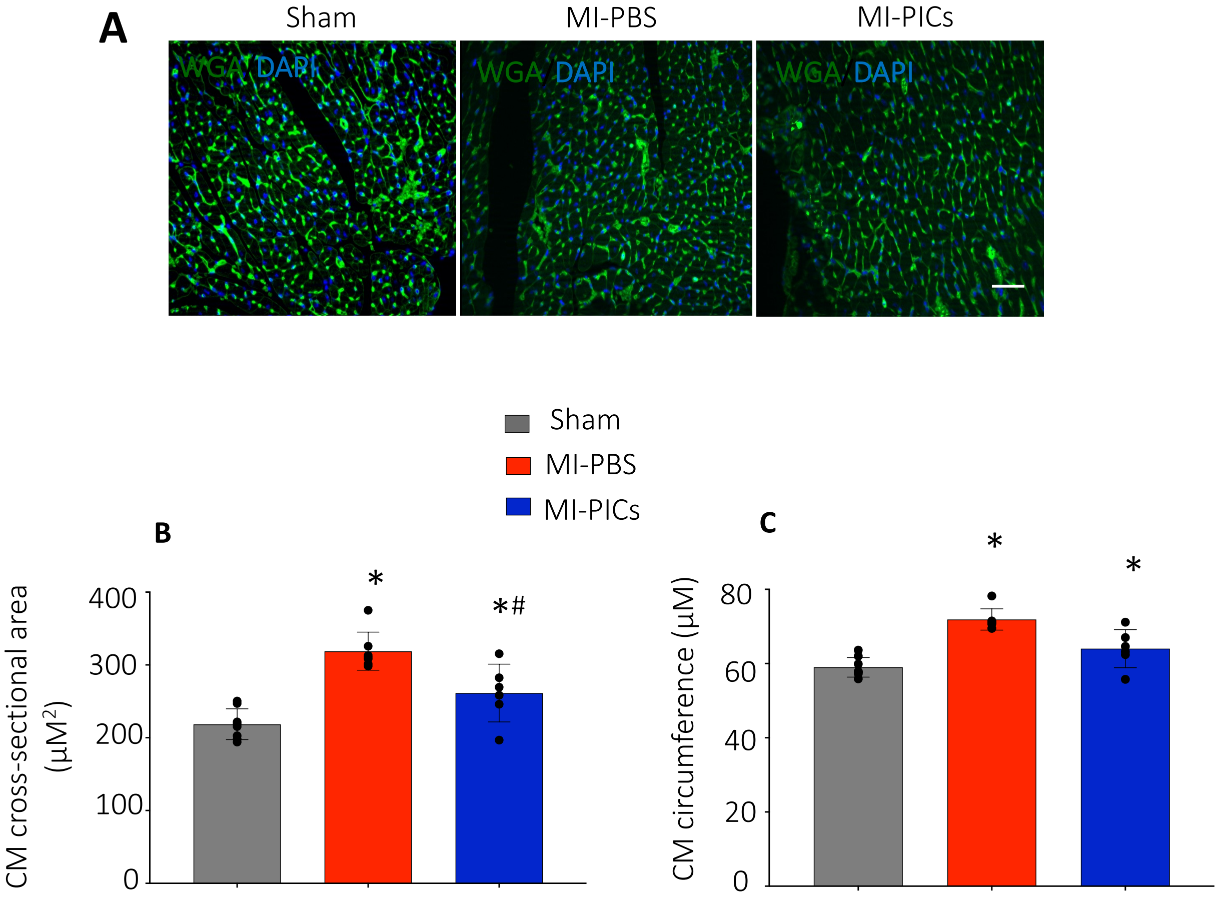

Supplementary Figure S2. A- Confocal images depicts hypertrophic cardiomyocytes in the non-RR of sham = 8, MI-PICs = 6 and MI-PBS = 7 groups, scale bar = 500 µM. WGA (green) and DAPI (blue). Measurements of the cross-sectional area, **B** and circumference, **C** of cardiomyocytes in the non-RR of the LV,. Data expressed as means ± SD *<0.05 vs. sham, #<0.05 vs MI-PBS.

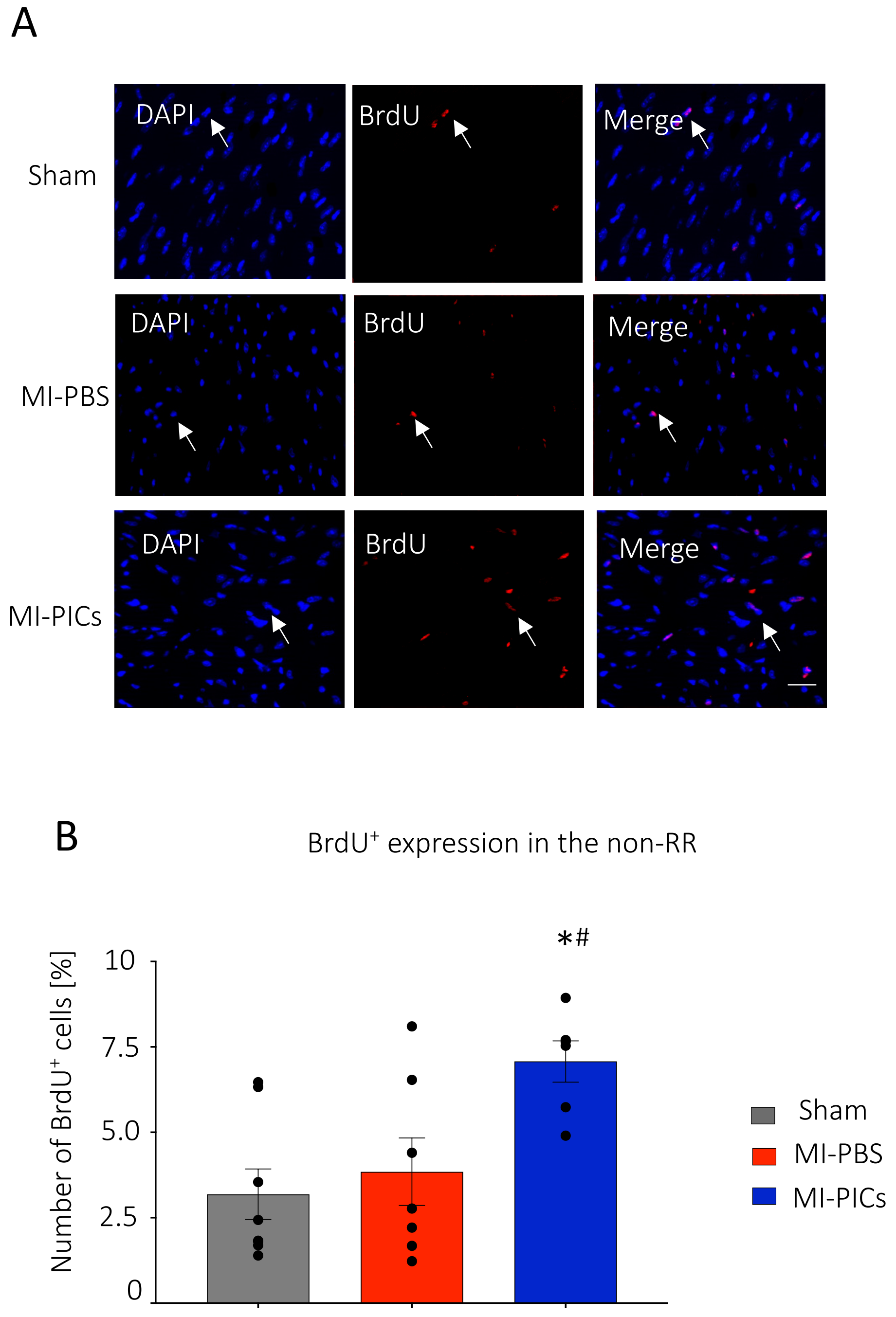

Supplementary Figure S3. Representative photomicrographs of the proliferation index in the non-RR of the LV in the sham = 8, MI-PBS = 7 and MI-PICs = 6 groups, Scale bar 10μM **A**. Quantification of the proliferating index in each group **B**, data expressed as means ± sem of the total number of BrdU$^{(+)}$ cells over the total number of cells (Dapi), *<0.05 vs. sham, #<0.05 vs MI-PBS.

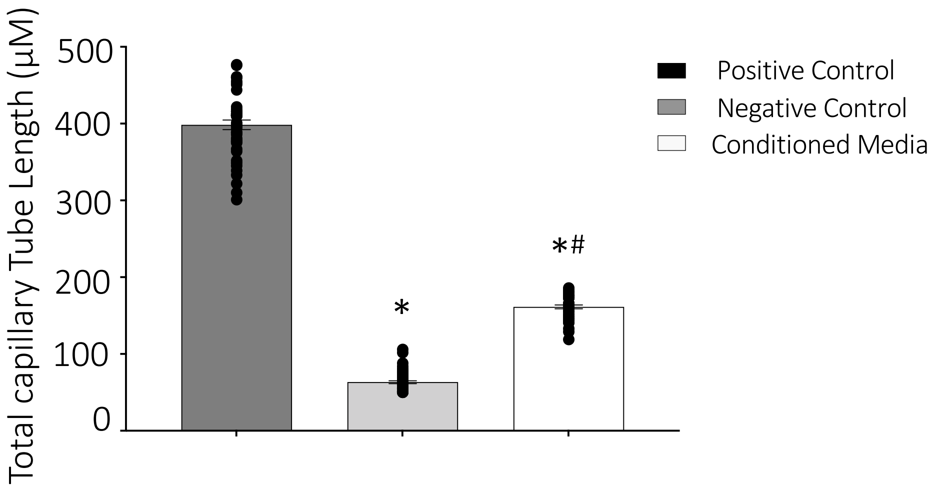

Supplementary Figure S4. Total capillary tube length as measured using a Matrigel tube formation assay. HUVECs were exposed for 15 h in 96-well plates coated with Matrigel to HUVEC media (positive control), serum-free DMEM (negative control) or PIC conditioned media. Data are presented as the Mean ± SEM. *<0.05 vs. Positive control, #<0.05 vs Negative control.
